# Cholesterol restricts lymphotoxin β receptor-triggered NF-κB signaling

**DOI:** 10.1186/s12964-019-0460-1

**Published:** 2019-12-26

**Authors:** Magdalena Banach-Orłowska, Renata Wyszyńska, Beata Pyrzyńska, Małgorzata Maksymowicz, Jakub Gołąb, Marta Miączyńska

**Affiliations:** 1 Laboratory of Cell Biology, International Institute of Molecular and Cell Biology, 02-109 Warsaw, Poland; 2 Department of Immunology, Medical University of Warsaw, Warsaw, Poland

**Keywords:** Lymphotoxin β receptor, Cholesterol, NF-κB signaling, NEMO, TRAF2, Receptor internalization

## Abstract

**Background:**

Lymphotoxin β receptor (LTβR) plays important roles in the development of the immune system and immune response. At the cellular level, ligand-bound LTβR activates the pro-inflammatory NF-κB pathway but the detailed mechanisms regulating its signaling remain unknown. Understanding them is of high importance since LTβR and its ligands are promising therapeutic targets. Here, we studied the consequences of perturbed cellular cholesterol content on LTβR-induced NF-κB signaling.

**Methods:**

To modulate cholesterol availability and/or level in lung carcinoma A549 and H2228, and endothelial HUVEC cells different treatment regimens with filipin, methyl-β-cyclodextrin and simvastatin were applied. LTβR localization was studied by confocal microscopy. The activity of LTβR-induced NF-κB pathway was assessed by measuring the levels of NF-κB pathway inhibitor IκBα and phosphorylation of RelA transcription factor by Western blotting. The NF-κB transcriptional response, production of chemokines and adhesion molecules were examined by qRT-PCR, ELISA, and Western blotting, respectively. Adherence of different types of primary immune cells to epithelial A549 cells and endothelial HUVECs was measured fluorometrically. Interactions of LTβR with its protein partners were investigated by immunoprecipitation.

**Results:**

We showed that filipin-mediated sequestration of cholesterol or its depletion from the plasma membrane with methyl-β-cyclodextrin impaired LTβR internalization and potentiated LTβR-dependent activation of the canonical branch of the NF-κB pathway. The latter was manifested by enhanced degradation of IκBα inhibitor, elevated RelA phosphorylation, substantial increase in the expression of NF-κB target genes encoding, among others, cytokines and adhesion molecules known to play important roles in immune response. It was followed by robust secretion of CXCL8 and upregulation of ICAM1, that favored the adhesion of immune cells (NK and T cells, neutrophils) to A549 cells and HUVECs. Mechanistically, we showed that cholesterol depletion stabilized interactions of ligand-stimulated LTβR with modified forms of TRAF2 and NEMO proteins.

**Conclusions:**

Our results showed that the reduction of the plasma membrane content of cholesterol or its sequestration strongly potentiated signaling outcome initiated by LTβR. Thus, drugs modulating cholesterol levels could potentially improve efficacy of LTβR-based therapies.

**Video abstract.**

## Background

Receptors belonging to the tumor necrosis factor receptor superfamily (TNFRSF) and their ligands have been exploited as promising therapeutic targets in the treatment of cancer and autoimmune diseases [1–3]. Lymphotoxin β receptor (LTβR) is a member of TNFRSF that binds lymphotoxin α1β2 and LIGHT (homologous to **l**ymphotoxin, exhibits **i**nducible expression and competes with HSV **g**lycoprotein D for binding to **h**erpesvirus entry mediator, a receptor expressed on **T** lymphocytes) [4, 5]. This receptor regulates a number of important processes including development of secondary lymphoid organs, such as Peyer’s patches [6] and lymph nodes [7], development of natural killer (NK) cells [8, 9], compartmentalization of dendritic cells [10] and T cell afferent lymphatic migration [11]. Due to the link between LTβR and immunity, LTβR and its ligands serve as an attractive target in treatment of various immunopathologies. Blocking of LTβR signaling was shown to exert beneficial effects in an experimental treatment of glomerulonephritis [12], Sjögren’s syndrome [13, 14], arthritis, diabetes, inflammatory bowel disease (reviewed in [15]) or in human rheumatoid arthritis (preclinical trials [1];). On the other hand, stimulation of LTβR-dependent pathways with LIGHT or agonistic antibody against LTβR promoted T cells infiltration into tumor, restricting its growth [16, 17].

LTβR triggers several signaling cascades. Its stimulation leads to the activation of NF-κB- and AP-1-dependent transcription [18, 19], and promotes apoptosis [20, 21]. The best characterized is the role of LTβR in the activation of the NF-κB pathway that operates via NF-κB1 (p50-RelA) and NF-κB2 (p52-RelB) transcription factors, within two branches – canonical and non-canonical, respectively [6, 18]. At the molecular level, the ligand binding leads to LTβR oligomerization and recruitment of adaptor proteins, tumor necrosis factor receptor associated factors (TRAF): TRAF2 and TRAF3 [20, 22] that occurs within minutes. It leads to the activation of an “immediate” – canonical branch of NF-κB pathway and a “delayed” non-canonical branch. Based on the data from studies on canonical NF-κB signaling activated by the founder of TNFRSF - tumor necrosis factor receptor (TNFR), binding of TRAF proteins to the cytoplasmic tail of the receptor is followed by recruitment of IκB kinase (IKK) complex [23] consisting of catalytic IKKα and IKKβ subunits and regulatory IKKγ, also known as NEMO (NF-κB essential modulator). Phosphorylation of IKKβ [24, 25] and polyubiquitylation of NEMO [26] enhances the activity of IKK complex responsible for the phosphorylation of the key pathway inhibitor IκBα that is prerequisite for its proteasomal degradation. In consequence, IκBα-bound NF-κB1 dimers are released and relocate to the nucleus where they bind to regulatory cis regions in DNA [27]. In contrast to TNFR, LTβR is able to activate also the non-canonical branch of the NF-κB pathway [28] that involves the activation of NF-κB-inducing kinase (NIK) and IKKα, which phosphorylate the inhibitor p100. Then p100 is polyubiquitylated and processed in a proteasome-dependent manner into p52 that together with RelB drives transcription of target genes [29, 30].

The signaling outputs initiated by receptors can be regulated by different factors, including local lipid composition of the surrounding membrane. The role of cholesterol, a crucial element of biological membranes, was shown in regulation of cellular signaling mediated by epidermal growth factor (EGF), insulin/IGF1, or neurotrophin receptors, and members of TNFRSF [31–34]. According to one study, relocation of ligand-bound TNFR1 to lipid rafts, the plasma membrane (PM) micro-domains enriched with cholesterol, was crucial for the NF-κB pathway activation [35], whereas another study proposed that lipid raft-localized TNFR1 activated ERK2, but not NF-κB [36]. Disruption of cholesterol-rich domains redirected TNFR1-dependent signaling from NF-κB towards pro-apoptotic cascades [35]. Cholesterol depletion significantly reduced the ability of another member of TNFRSF - death receptor 5 (DR5) to initiate apoptosis [37], indicating the role of cholesterol-rich micro-domains in pro-apoptotic signaling initiated by DR5 ligand TRAIL in TRAIL-sensitive non-small-cell lung carcinoma (NSCLC) cells [38]. Interestingly, the PM domains other than lipid rafts were important for TRAIL-dependent activation of NF-κB and ERK1/2 in TRAIL-resistant NSCLC cells [38].

Manipulations in the cholesterol level were considered as a therapeutic strategy. In ErbB2-positive breast cancer lovastatin, a cholesterol-lowering drug sensitized cancer cells to lapatinib and neratinib [34]. Depletion of the PM cholesterol by methyl-β-cyclodextrin (MβCD) was proposed as a tool in the treatment of synucleinopathies [39] or melanoma [40]. In T24 high-grade invasive urothelial cancer cells MβCD induced cell death [41]. Cyclodextrins are also considered as an effective tool to interfere with atherosclerosis pathogenesis [42].

Despite vast knowledge about a physiological role of LTβR, there is still a gap in understanding the mechanisms regulating its signaling at the cellular level. Thus identifying factors influencing the LTβR activity could create an opportunity to develop novel therapeutic strategies. Here, we reveal that cholesterol depletion activates LTβR-triggered canonical branch of the NF-κB pathway that could constitute a potential strategy to improve LTβR-based therapies.

## Methods

### Cell lines

A549 cells were purchased from Sigma-Aldrich, H2228 and Jurkat cells were purchased from ATCC and later authenticated as required. Cells were maintained in Dulbecco’s modified Eagle’s medium (DMEM) high glucose (Merck) or RPMI-1640, respectively. Media were supplemented with 10% fetal bovine serum (FBS) and 2 mM L-glutamine (Merck), where required. Cells were routinely tested for mycoplasma contamination. HUVECs were purchased from PromoCell and cultured in Endothelial Cell Growth Medium 2 with Supplement Mix according to manufacturer’s guidelines.

### Antibodies and other reagents

Primary antibodies used for Western blotting are listed in Additional file 2: Table S1.

Secondary antibodies: horseradish peroxidase-conjugated anti-rabbit (111–035-144), anti-mouse (111–035-062), and anti-goat (805–035-180) antibodies were purchased from Jackson ImmunoResearch; secondary fluorophore-conjugated anti-mouse IRDye 800CW (926–32212) antibodies for the Odyssey system were from LICOR Biosciences. All secondary antibodies for Western blotting were diluted 1:10,000.

Primary antibodies used for immunofluorescence are listed in Additional file 2: Table S2.

Secondary antibodies used for immunofluorescence: Alexa Fluor 488-, 555-, 647-conjugated anti-goat, anti-mouse and anti-rabbit (Thermo Fisher Scientific) were diluted 1:500.

For immunoprecipitation, goat agonistic anti-LTβR (AF629, R&D Systems) and control goat IgG (I5256, Sigma-Aldrich) were used.

Anti-LTβR agonistic antibody (AF629, R&D Systems) and human recombinant lymphotoxin α1β2 (8884-LY-025/CF, R&D Systems) were used at concentration 0.2 μg/ml. MβCD (C4555, Merck) was used at 2.5 or 5 mM concentration in medium without FBS. Simvastatin (S6196, Merck) was used at 30 μM concentration for 48 h in medium supplemented with delipidated FBS (S181 L, Biowest). Filipin III (F4767, Merck) was used at a concentration of 1 μg/ml medium without FBS. Cholesterol (C3045, Merck) was dissolved in ethanol and then complexed with MβCD to concentration (50 mM MβCD: 5 mM Cholesterol). Final concentration of MβCD: Cholesterol complex was 2.5 mM: 0.25 mM, respectively. TAK-243 (HY-100487, MedChemExpress) was used at 1 μM concentration (diluted in DMSO) in medium without FBS for 5.5 h in total (4 h pre-incubation followed by 1 h treatment with vehicle or MβCD and 0.5 h stimulation with lymphotoxin α1β2).

### Cholesterol sequestration/depletion experiments

Two days before experiment cells were seeded on 24-, 12-, 6-well plate or 10 cm dishes (4.5 × 10^4^, 10 × 10^4^, 25 × 10^4^, 200 × 10^4^, respectively) depending on a type of assay (microscopy, Western blotting/ qRT-PCR, immunoprecipitation). At the days of experiments cells were washed twice with PBS to remove exogenous lipids.

Cholesterol sequestration was performed using filipin III (F4767, Merck) at 1 μg/ml concentration in medium without serum. To minimize toxic effects of filipin, antibiotic was administered only for 0.5 h pretreatment. Following stimulation with LTβR agonist was carried out in the absence of filipin for next 0.5 h and 1 h.

Acute cholesterol depletion was performed using MβCD (C4555, Merck) at a concentration of 5 mM (short treatments – 0.5, 1 or 4 h) or 2.5 mM (long treatment – 6, or 8 h).

Chronic cholesterol depletion was achieved by incubating the cells in delipidated medium containing 30 μM simvastatin (S6196, Merck) for 48 h. Then LTβR stimulation was carried out in the same medium supplemented with LTβR agonist or lymphotoxin α1β2.

### Cholesterol replenishment experiments

Cholesterol replenishment experiments were performed as summarized on the scheme presented in Fig. 3a). In more details: cells were plated 2 days before experiment as described above. At the day of experiment cells were washed twice with PBS and pretreated with 5 mM MβCD (in medium without FBS) for 1 h. Then the media was exchanged for add-back medium containing cholesterol complexed with MβCD (final concentration was 2.5 mM MβCD: 2.5 mM cholesterol) or control media: 2.5 mM MβCD supplemented with ethanol or with appropriate volume of water and ethanol solutions.

After 0.5 h of cholesterol replenishment, the media were exchanged for the same, but supplemented with LTβR agonist. Stimulation was performed for 0.5 and 1 h. Then cells were lysed or fixed and analyzed by Western blotting or microscopy, respectively.

### Western blotting

Cells were lysed in RIPA buffer (1% Triton X-100, 0.5% sodium deoxycholate, 0.1% SDS, 50 mM Tris pH 7.4, 150 mM NaCl, 0.5 mM EDTA) or in buffer for immunoprecipitation (IP buffer: 50 mM HEPES, pH 7.5, 150 mM NaCl, 1 mM EGTA, 1 mM EDTA, 1% Triton X-100, 10% glycerol), supplemented with protease inhibitor cocktail (6 μg/ml chymostatin, 0.5 μg/ml leupeptin, 10 μg/ml antipain, 2 μg/ml aprotinin, 0.7 μg/ml pepstatin A and 10 μg/ml 4-amidinophenylmethanesulfonyl fluoride hydrochloride; Sigma-Aldrich) and phosphatase inhibitor cocktails (P0044 and P5726, Sigma-Aldrich). Protein concentration was assessed with BCA Protein Assay Kit (Thermo Fisher Scientific). Then, 25–30 μg of total protein/sample were resolved on 10–14% polyacrylamide gels, transferred to nitrocellulose membrane (Whatman), that was incubated with specific primary and secondary antibodies. For signal detection either ChemiDoc imaging system (Bio-Rad) or Odyssey infrared imaging system (LI-COR Biosciences) were employed. Densitometry analysis of protein bands was performed using ImageJ Software [43].

### Immunofluorescence staining and image analysis

Cells upon treatment were transferred to ice, washed twice with ice-cold PBS, and fixed with ice-cold 3.6% paraformaldehyde for 15 min. After three washes with PBS, cells were immunostained, as described previously [44–46].

Slides were scanned using a ZEISS LSM 710 confocal microscope with EC Plan-Neofluar 40 × 1.3 NA oil immersion objective. ZEN 2009 software (Zeiss) was used for image acquisition. At least ten 12-bit images with resolution 1024 × 1024 pixels were acquired per experimental condition. Images were then analyzed by MotionTracking software (http://motiontracking.mpi-cbg.de) with respect to integral intensity and number of LTβR- and EEA1-positive vesicles [47–49]. Images were then assembled in Photoshop (Adobe) with only linear adjustments of contrast and brightness.

### Transfection with small interfering RNA (siRNAs)

siRNA reverse transfections were performed using RNAiMAX (Thermo Fisher Scientific) according to manufacturers’ instructions. For microscopic assays 3 × 10^4^ cells/well were put on 12 mm coverslips in a 24-well plate; for Western blotting 6 × 10^4^ cells/well were put in 12-well plate. Cells were analyzed 72 h post transfection. The concentration of siRNA was 20 nM. siRNAs (Ambion Silencer Select; Thermo Fisher Scientific) used in this study: Ctrl_1 (Negative Control No. 1, 4,390,843), Ctrl_2 (Negative Control No. 2, 4,390,846), Caveolin-1_1 (s2446; GCUUCCUGAUUGAGAUUCAtt), Caveolin-1_2 (s2448; CCUUCACUGUGACGAAAUA), Cavin-1_1 (s49508; CGAGCAAUACGGUGAGCAAtt), Cavin-1_2 (s49507; CAUCUCUACUAAGCGAAAAtt), TRAF2_1 (s14381; UUCAAUCUUGUCUUGGUCCag), TRAF2_2 (s14380; ACAAGUCUUGACGUGGUCCtg).

### Generation of LTβR knock-out A549 cell line clones

Knock-out of LTβR in A459 cells was performed using CRISPR/Cas9 technology as described before [50]. Two 25-bp-long single guide RNA (sgRNAs) were designed based on Brunello library [51](Additional file 2: Table S3) and cloned into LentiCRISPR v2 vector (Addgene vector #52961). Plasmids encoding non-targeting sgRNA (kind gift from Dr. Katarzyna Mleczko-Sanecka) were designed based on [52].

Production of lentiviruses and infection of A549 cells were performed according to protocol described before [50]. After ten days of selection for puromycin (1.2 μg/ml) resistance, cells were plated in medium without antibiotic and analyzed for knock-out efficiency. Then clonal selection was performed. For each sgRNA four clones with complete knock-out were selected and pooled. Pools of clones (equal number of cells of each clone was combined) were used for experiments.

### Quantitative real-time PCR (qRT-PCR)

Total RNA was isolated with High Pure Isolation Kit (11828665001, Roche). For cDNA synthesis M-MLV reverse transcriptase, random nonamers and oligo (dT)_23_ (Sigma-Aldrich) were used according to manufacturer’s instructions.

To estimate the expression of genes of interest we performed the qRT-PCR reaction with primers designed with NCBI tool (and custom-synthesized by Sigma-Aldrich) (listed in Additional file 2: Table S4) or TaqMan® Gene Expression Assays (Thermo Fisher Scientific) (listed in Additional file 2: Table S5).

For the qRT-PCR reaction we used the KAPA SYBR FAST qPCR Master Mix (2X) Universal Kit (KK4618, KapaBiosystems) or TaqMan® Gene Expression Master Mix (4369016, ThermoFisher Scientific) and a 7900HT Fast Real-Time PCR thermocycler (Applied Biosystems). At least two technical repeats per experimental condition were done. The expression of targets were normalized to the level of housekeeping genes *ACTB*, *B2M,* and *GAPDH* and presented as fold changes.

### Immunoprecipitation (IP)

A549 cell lysates were prepared in the immunoprecipitation buffer (IP buffer), supplemented with protease and phosphatase inhibitor cocktails. From 250 to 500 μg of protein was used per pull-down. Cell extracts were diluted in IP buffer and pre-cleared for 2 h at 4 °C with goat IgG (Sigma Aldrich) and Protein G agarose beads (Roche) to deplete nonspecifically bound proteins. Then, cell lysates were incubated overnight at 4 °C (with constant agitation) with appropriate amounts of antibodies (1.25 μg of antibodies/500 μg of total protein), specific anti-LTβR or unspecific - control goat IgG or specific anti-TRAF2 or control mouse IgG. Immune complexes were recovered by incubation with Protein G–agarose beads at 4 °C with agitation for 2 h. The protein complexes bound to agarose beads were spun down and washed five times with IP buffer. Next, samples were incubated at 95 °C for 10 min with Laemmli buffer and subjected to electrophoresis on 10% polyacrylamide gels.

### Elisa

Cytokine levels were measured in cell culture medium using Human IL-8 ELISA Kit (ab214030, Abcam) accordingly to the manufacturer’s instructions. Colorimetric measurements were performed on the Sunrise Plate Reader (TECAN). All samples and standards were measured in duplicates.

### Isolation of the immune cells

Neutrophils were isolated from 10 ml of fresh samples of whole peripheral blood from healthy donors using EasySep Direct Human Neutrophil Isolation Kit (19666, STEMCELL Technologies), accordingly to the manufacturer’s instructions. In addition, buffy coats of healthy donors were used for isolation of peripheral blood mononuclear cells (PBMCs) with Lymphoprep density gradient medium (07851, STEMCELL Technologies). Approval for the study was obtained from the Institutional Review Board of the Medical University of Warsaw. NK and T cells were isolated from PBMCs using EasySep Human NK Cell Enrichment Kit (19055, STEMCELL Technologies) and EasySep Human T Cell Isolation Kit (17951, STEMCELL Technologies), respectively.

### Adhesion assay

A549 cells and HUVECs were seeded in a black 96-well plate with transparent bottoms (655090, Greiner bio-one) at 5–10 × 10^4^ cells per well, respectively in complete media 2 days before the assay. On the day of the assay cells were washed with PBS, pretreated for 1 h with 2.5 mM MβCD and stimulated or not for 8 (A549) or 6 h (HUVECs) with LTα1β2 in the presence of 2.5 mM MβCD or vehicle in medium without serum. On the same day, immune cells were stained with CFSE (65–0850, Thermo Fisher Scientific) accordingly to the manufacturer instructions. The stained immune cells were re-suspended in RPMI or DMEM medium w/o serum (8 × 10^5^ cells/ml) and 100 μl of cell suspension were loaded on A549 cells or HUVECs treated as described above. After 35 min of co-culture non-adherent immune cells were extensively washed away with serum-free DMEM medium. Fluorescence was measured with the Infinite M1000 Plate Fluorimeter (TECAN) using 492/517 nm excitation/emission filter sets. Each condition was tested in duplicates or triplicates.

### Statistical analysis

Each type of experiment was performed at least 3 times. For statistical analysis Prism 6 (GraphPad Software) was used. Data were analyzed for Gaussian distribution with a Kolmogorov-Smirnov test. In case of Gaussian distribution, the following parametric tests were used: Student’s t-test or one-way ANOVA (with Dunnett’s post-hoc test), as appropriate. In case of non-Gaussian distribution Mann-Whitney (with Dunn’s post-hoc test) was used. To assess the significance of differences in fold changes vs control set as 1 we used one sample t-test. The significance of mean comparison is annotated as follows: ns, non-significant (*P* > 0.05), *P ≤ 0.05, **P < 0.01, and ***P ≤ 0.001.

## Results

### Sequestration of cholesterol augments LTβR-dependent NF-κB signaling and impairs LTβR internalization

Previous studies revealed that human lung carcinoma A549 cells are suitable to study LTβR signaling in vitro [44, 53]. In response to LTβR ligation these cells activate canonical NF-κB signaling, that is manifested by degradation of the pathway inhibitor IκBα and phosphorylation of RelA at Ser536 (P-RelA) with the kinetics similar to that observed in other cell lines [54, 55]. Upon 0.5–1 h of LTβR stimulation with agonistic anti-LTβR antibody (Ago) or lymphotoxin α1β2 (LTα1β2) we observed a decreased amounts of IκBα, that returned to the basal levels within 1.5 h, while phosphorylation of RelA was increased through the whole stimulation period (Additional file 1: Figure S1a, b).

**Fig. 1:**
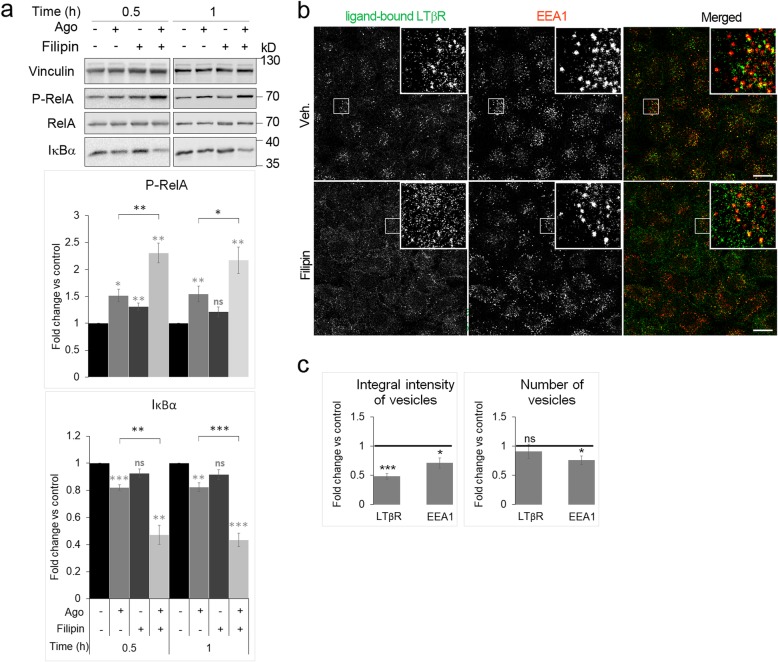
Sequestration of the plasma membrane pool of cholesterol by filipin enhances LTβR-triggered activity of the NF-κB pathway and reduces internalization of the ligand-bound receptor. **a** Lysates of A549 cells preincubated for 0.5 h with filipin or vehicle and stimulated for 0.5 or 1 h with LTβR agonist (Ago) were analyzed by Western blotting with antibodies against the indicated proteins. Vinculin was used as a loading control. Graphs show densitometric analysis for the indicated proteins from Western blotting (protein levels normalized to vinculin). Values are presented as fold change versus controls - unstimulated and untreated cells (black bars). Data represent the means ± SEM, *n* ≥ 5; ns - *P* > 0.05; *P ≤ 0.05; **P ≤ 0.01; ***P ≤ 0.001 by one sample t-test (in grey), Student’s t*-*test (in black). **b** Immunofluorescence staining of ligand-bound LTβR and EEA1 upon 0.5 h stimulation with LTβR agonist in A549 cells preincubated for 0.5 h with either vehicle (Veh.) or filipin. Insets: magnified views of boxed regions in the main images. Scale bars, 20 μm. **c** Analysis of integral intensity and number of LTβR- and EEA1-positive vesicles in cells treated as in B. Values are presented as fold change versus control - vehicle-treated cells marked as a black line, set as 1. Data represent the means ± SEM, *n* = 5. ns - *P* > 0.05; *P ≤ 0.05; ***P ≤ 0.001 by one sample t-test

To assess the role of cholesterol in the regulation of LTβR signaling, we measured the activity of the NF-κB pathway in cells with sequestered cholesterol. To this end, we incubated A549 cells with filipin, a polyene antibiotic that selectively binds to, and sequesters cholesterol in the PM [56, 57]. Taking into account the kinetics of LTβR–triggered canonical NF-κB signaling we measured its activity by assessing the status of its effector proteins in cells pretreated with filipin and then stimulated with Ago for short time periods (0.5 and 1 h). Filipin alone had no effect on the levels of IκBα and a minor and transient one (only at the 0.5 h) on the phosphorylation of RelA, whereas sequestration of cholesterol by filipin significantly enhanced the NF-κB pathway activation caused by Ago (Fig. 1a). This synergistic effect was observed for both IκBα and P-RelA levels.

As filipin impairs caveolae-dependent endocytosis [58, 59], we examined its effects on LTβR internalization. To this end, we used confocal microscopy to measure the amounts of ligand-bound receptor on vesicular structures upon 0.5 h stimulation with Ago in controls and in cells with cholesterol sequestered by filipin. Stimulation of cells with Ago followed by immunostaining with a secondary antibody recognizing Ago allowed tracking specifically a ligand-bound pool of the receptor. We quantitatively analyzed the microscopic images with respect to integral fluorescence intensity of LTβR- as well as EEA1-positive vesicles (early endosomes). Integral intensity is a parameter which reflects the amounts of a particular protein in vesicular structures (Fig. 1b). We noticed a substantial (> 50%) reduction of integral intensity of LTβR-positive vesicles and only a minor decrease of integral intensity of EEA1-positive vesicles in cells pretreated with filipin (Fig. 1c). The number of LTβR vesicles was not reduced upon cholesterol sequestration (Fig. 1c). It suggested that endosomes harboring LTβR are still formed but loaded with lower amounts of the receptor that is reflected by the decrease in integral intensity of LTβR-positive structures. These data indicate that cholesterol-dependent internalization of LTβR inhibits NF-κB signaling.

### Pharmacological inhibition of cholesterol synthesis activates NF-κB signaling in LTβR-independent manner

To confirm that LTβR signaling is regulated by cholesterol, we performed its chronic depletion. We cultured A549 cells for 48 h in medium containing simvastatin that inhibits HMG-CoA reductase – a key enzyme of the cholesterol biosynthesis pathway [60], in the absence of exogenous source of cholesterol. Similarly to the analysis described above, we measured the activity of canonical NF-κB signaling in simvastatin-treated cells stimulated with Ago for short time periods (0.5 and 1 h). We found that simvastatin alone increased RelA phosphorylation as well as enhanced the degradation of IκBα. At the same time, cells treated with both simvastatin and Ago did not exhibit further enhancement of RelA phosphorylation, whereas the degradation of IκBα was potentiated upon 1 h of stimulation (Additional file 1: Figure S2a).

Next, we investigated the internalization of ligand-bound receptor upon stimulation with Ago as described above. We noticed a significant decrease of ligand-bound LTβR internalization in cells treated with simvastatin that was manifested by the reduction of both integral intensity and the number of vesicles marked with LTβR (Additional file 1: Figure S2b). Simvastatin caused general changes in the endocytic system, as demonstrated by an increase of integral intensity and the number of EEA1-positive vesicles (early endocytic compartments). We also examined the intracellular pool of the receptor by immunostaining of LTβR using Ago as a primary antibody in unstimulated cells. We noticed a substantial increase of total amounts of the receptor in cells treated with simvastatin (Additional file 1: Figure S2c) that was confirmed by biochemical approaches (Additional file 1: Figure S2d). The elevated levels of the protein were not caused by activation of *LTΒR* gene transcription as we did not found significant changes at the mRNA level (Additional file 1: Figure S2e).

Since the intracellular accumulation of LTβR can stimulate the NF-κB pathway in a ligand-independent manner [44], we checked if the effect of simvastatin on NF-κB signaling depended on LTβR. To this end we generated LTβR knock-out A549 cell line clones using the CRISPR/Cas9 technology and treated them with simvastatin. We found that inhibition of cholesterol synthesis activated the NF-κB pathway to the same extent in the presence and the absence of LTβR in the cell (Additional file 1: Figure S3).

Altogether, these data allowed us to conclude that inhibition of cholesterol biosynthesis by simvastatin impairs the intracellular trafficking of LTβR, affects the NF-κB pathway activity, independently of LTβR and exerts pleiotropic effects such as global changes in the endocytic machinery.

### Acute depletion of cholesterol augments LTβR-dependent NF-κB signaling

**Fig. 2:**
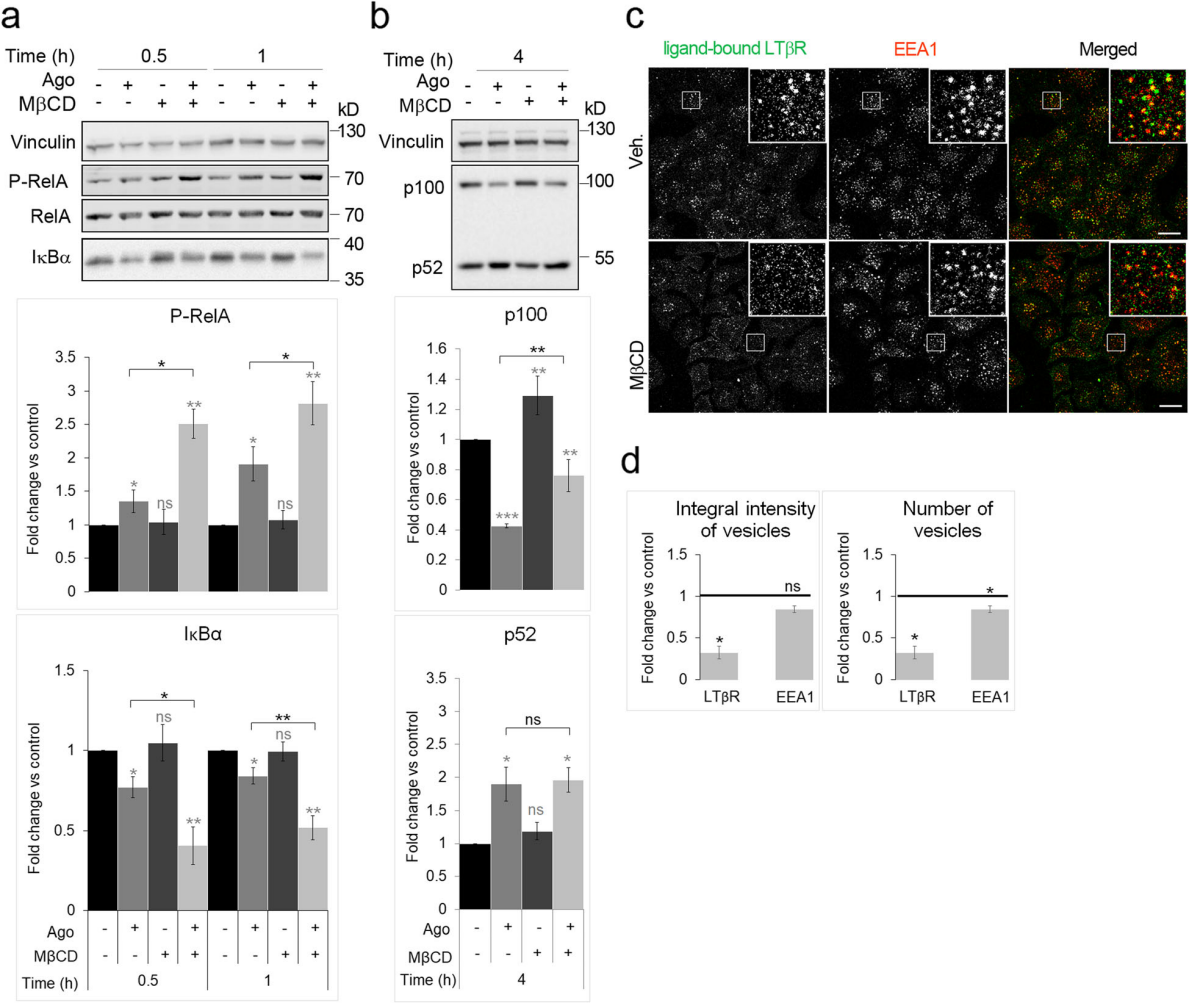
Cholesterol depletion by MβCD enhances LTβR-triggered activity of the NF-κB pathway and reduces internalization of the ligand-bound receptor. **a, b** Lysates of A549 cells preincubated for 1 h with MβCD or vehicle and stimulated for 0.5, 1 or 4 h with LTβR agonist (Ago) were analyzed by Western blotting with antibodies against the indicated proteins. Vinculin was used as a loading control. Graphs show densitometric analysis for the indicated proteins from Western blotting (protein levels normalized to vinculin). Values are presented as fold change versus controls - unstimulated and untreated cells (black bars). Data represent the means ± SEM, *n* = 6 (a), *n* = 4 (b); ns - *P* > 0.05; *P ≤ 0.05; **P ≤ 0.01 by one sample t-test (in grey) and Student’s t*-*test (in black). **c** Immunofluorescence staining of ligand-bound LTβR and EEA1 in A549 cells upon 0.5 h stimulation with LTβR agonist in cells preincubated with vehicle (Veh.) or MβCD. Insets: magnified views of boxed regions in the main images. Scale bars, 20 μm. **d** Analysis of integral intensity and the number of LTβR- and EEA1-positive vesicles in cells treated as in B. Values are presented as fold change versus control - vehicle-treated cells marked as a black line, set as 1. Data represent the means ± SEM, *n* = 3. ns - *P* > 0.05; *P ≤ 0.05 by one sample t-test

As an alternative to simvastatin-mediated inhibition of cholesterol synthesis, we performed a spatially restricted reduction of the cholesterol level at the PM. We employed methyl-β-cyclodextrin (MβCD), a reagent widely used to bind and extract cholesterol from membranes [57]. We measured the activity of both: canonical and non-canonical NF-κB pathways in cells stimulated with Ago for short (0.5 and 1 h) or long (4 h) time periods, respectively. We found that MβCD alone did not affect the activity of the canonical pathway, however it potentiated the pathway activation by Ago. This synergistic effect of MβCD and Ago on the degradation of IκBα as well as on RelA phosphorylation was clearly observed upon 0.5 and 1 h of LTβR stimulation (Fig. 2a). Conversely, MβCD did not enhance the activation of the non-canonical NF-κB pathway by LTβR as the processing of p100 to p52 in response to the stimulation with Ago (clearly detected upon 4 h of treatment) remained unchanged in the presence of MβCD (Fig. 2b).

Analogically to the experiments described above, we investigated internalization of ligand-bound LTβR upon 0.5 h stimulation with Ago in cholesterol-depleted cells (Fig. 2c). In comparison to normal conditions (in the presence of vehicle), treatment with MβCD substantially reduced both the integral intensity and the number of LTβR vesicles, whereas it did not affect the early endocytic compartment, as judged by EEA1 staining (Fig. 2d). In parallel we assessed the intracellular distribution of the receptor. We found that the integral intensity and the number of LTβR-positive vesicles remained unchanged upon incubation with MβCD (Additional file 1: Figure S4a, b). Of note, there were no changes in the amounts of EEA1 marker on the vesicles and in the number of early endosomes that confirmed data from Ago-stimulated cells.

**Fig. 3:**
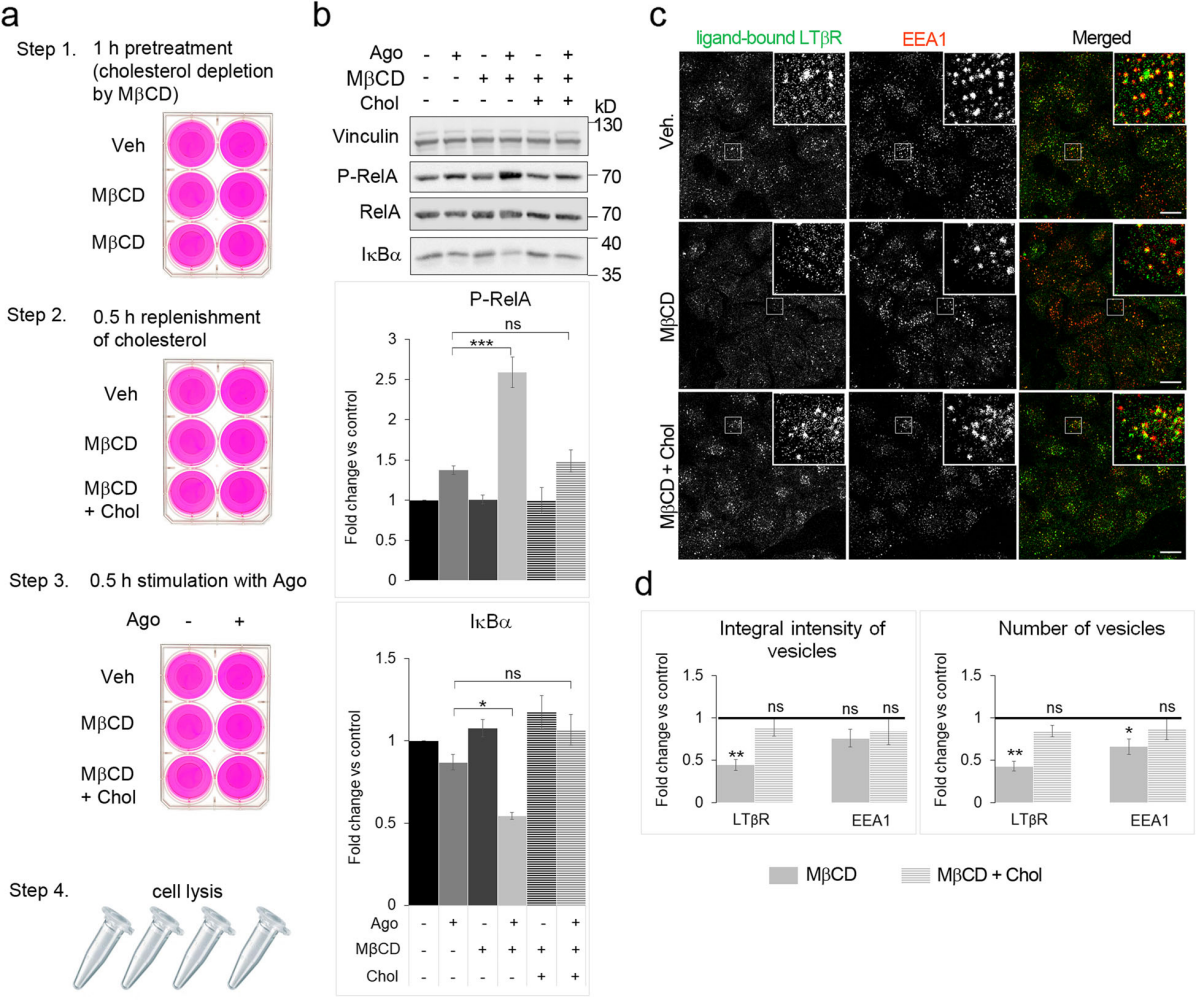
Cholesterol replenishment rescues the effects of MβCD treatment on LTβR signaling and internalization. **a** Schematic description of cholesterol replenishment experiments. **b** Lysates of A549 cells treated as depicted in a were analyzed by Western blotting with antibodies against the indicated proteins. Vinculin was used as a loading control. Graphs depict densitometric analysis for the indicated proteins from Western blotting (protein levels normalized to vinculin). Values are presented as fold change versus controls - unstimulated and untreated cells (black bars). Data represent the means ± SEM, n = 5; ns - *P* > 0.05; *P ≤ 0.05; ***P ≤ 0.001 by ANOVA test. **c** Immunofluorescence staining of ligand-bound LTβR and EEA1 in A549 cells treated as depicted in A except Step 4, where cells were fixed and stained instead of cell lysis. Insets: magnified views of boxed regions in the main images. Scale bars, 20 μm. **d** Analysis of integral intensity and the number of LTβR- and EEA1-positive vesicles in cells treated as in C. Values are presented as fold change versus controls - vehicle-treated cells marked as a black line, set as 1. Data represent the means ± SEM, n = 3. ns - *P* > 0.05; *P ≤ 0.05; **P ≤ 0.01 by one sample t-test

To see whether the effects observed upon MβCD treatment reflected specifically cholesterol depletion rather than off-target effects of MβCD, we performed add-back experiments with cholesterol replenishment, as shown in Fig. 3a. Briefly, cells preincubated with MβCD were challenged with cholesterol complexed with MβCD, that allowed “refilling” the PM with cholesterol. As a control we used cells treated with medium containing MβCD alone. Then cells were stimulated with Ago and examined with respect to NF-κB signaling. The obtained data clearly showed that cholesterol replenishment rescued the effects observed upon cholesterol depletion. The increased phosphorylation of RelA and the enhanced degradation of IκBα in MβCD-treated and Ago-stimulated cells returned to the level observed in Ago-stimulated cells not incubated with MβCD (Fig. 3b). Moreover, we examined the internalization of ligand-bound receptors upon cholesterol replenishment. We found that both integral intensity and the number of vesicles harboring LTβR increased to the levels observed in cells with unaffected cholesterol content (Fig. 3c, d).

All these observations allow concluding that the PM cholesterol depletion by MβCD does not affect the endocytic machinery in general, or the intracellular distribution of the receptor. It restricts the internalization of a ligand-bound pool of LTβR and potentiates LTβR-dependent activation of NF-κB signaling. Thus, we decided to continue our studies on LTβR signaling using MβCD, as a tool to alter the content of the PM cholesterol.

### Impairment of cavin-1-dependent LTβR internalization is insufficient to affect the signaling outcome of the receptor

As the observed changes in LTβR-triggered NF-κB signaling correlated with changes in receptor internalization, we checked if reduction of LTβR endocytosis would be sufficient to activate the NF-κB pathway. Specifically, we aimed to reduce endocytosis of the receptor and check its effect on LTβR-triggered NF-κB signaling. We silenced expression of genes encoding caveolin-1 and cavin-1 to block caveolae-dependent endocytosis, a route strongly dependent on cholesterol [61, 62]. We noticed that, despite a very good knock-down efficiency, caveolin-1 depletion did not affect the internalization of ligand-bound LTβR (Additional file 1: Figure S5a). However, depletion of cavin-1 reduced both integral intensity and the number of LTβR vesicles (Additional file 1: Figure S5b). Thus, we examined the NF-κB pathway activity in cavin-1-deprived cells and found that the responsiveness of cells to Ago measured by the degradation of IκBα upon 0.5 and 1 h of stimulation remained unchanged (Additional file 1: Figure S5c).

Cumulatively, these findings suggest that the reduction of LTβR internalization is not sufficient for sensitizing cells to Ago. Instead, the PM cholesterol content appears to influence LTβR-driven NF-κB signaling.

### Cholesterol depletion enhances interactions between LTβR and TRAF2 and NEMO proteins

**Fig. 4:**
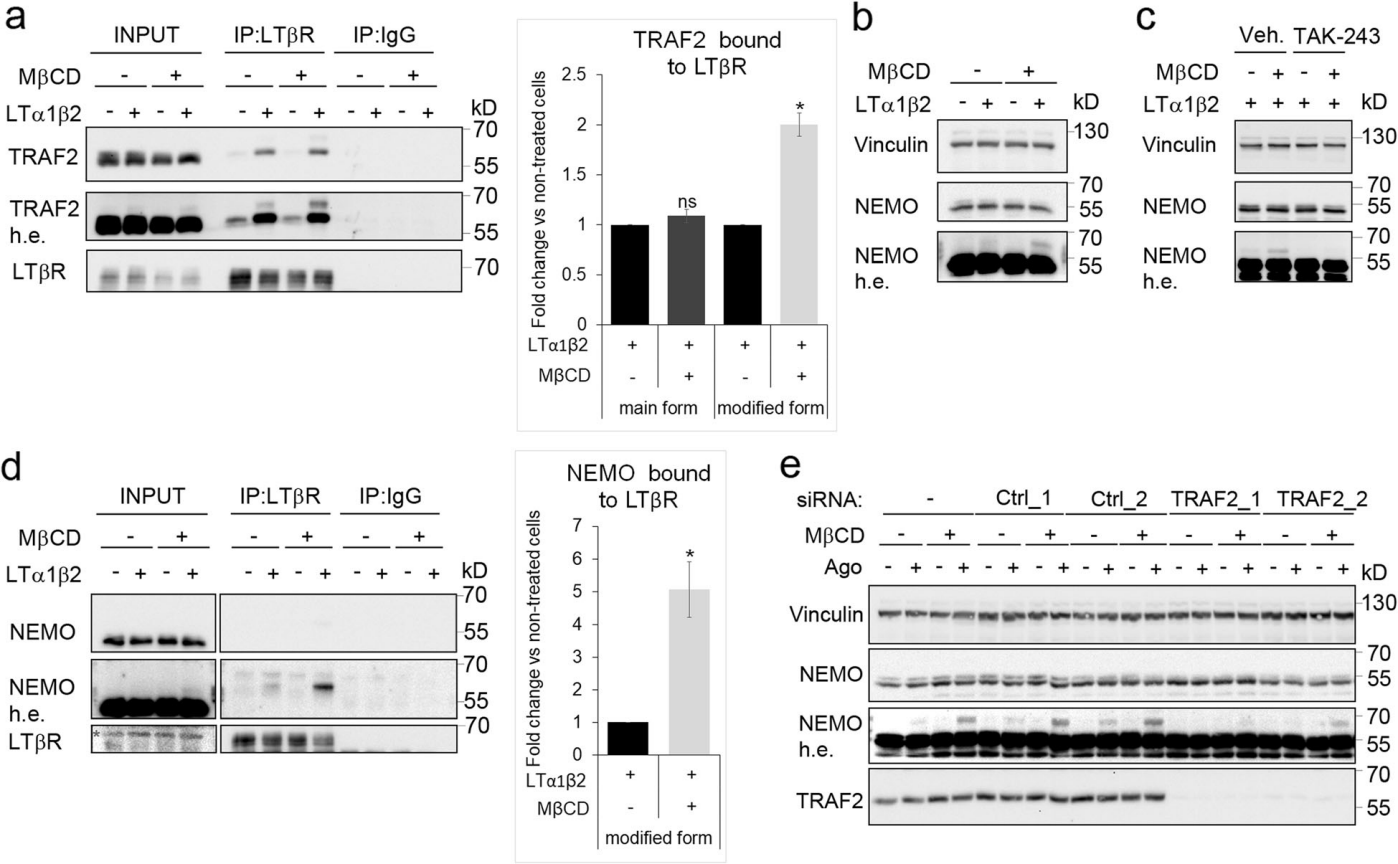
Cholesterol depletion enhances binding between LTβR and modified forms of TRAF2 and NEMO. **a** Western blot analysis of co-immunoprecipitates of anti-LTβR (IP:LTβR) and control antibodies (IP:IgG) from extracts of A549 cells stimulated with LTα1β2 for 0.5 h upon 1 h preincubation in medium containing either MβCD or vehicle. Antibodies against LTβR, TRAF2 were used for blotting. Input represents 10% of the lysates used for IP. h. e. – high exposure. Graph depicts the analysis of TRAF2 abundance (the main and modified forms of the protein) in LTβR co-immunoprecipitates upon stimulation with LTα1β2. The ratio of co-immunoprecipitated TRAF2 to immunoprecipitated LTβR was quantified. Data were normalized to the TRAF2-LTβR ratio in cells not treated with MβCD, which was assigned a value of 1. Data represent the means ± SEM, n = 3. ns - *P* > 0.05; *P ≤ 0.05 by one sample t-test. **b** Lysates of A549 cells preincubated for 1 h with MβCD and then stimulated or not for 0.5 h with LTα1β2 in the presence or the absence of MβCD were analyzed by Western blotting with antibodies against NEMO. h. e. – high exposure. Vinculin was used as a loading control. **c** Lysates of A549 cells preincubated for 4 h with TAK-243 or vehicle, treated or not for following 1 h with MβCD and then stimulated for 0.5 h with LTα1β2 were analyzed by Western blotting with antibodies against NEMO. h. e. – high exposure. Vinculin was used as a loading control. **d** Western blot analysis of immunoprecipitation performed as in A. Antibodies against LTβR and NEMO were used for blotting. Input represents 5% of lysates used for IP. Graph shows the abundance of modified NEMO in LTβR immunoprecipitates. Asterisk marks an unspecific band recognized by anti-LTβR antibody. Quantification as in A. Data represent the means ± SEM, n = 3. ns - *P* > 0.05; *P ≤ 0.05 by one sample t-test. **e** Lysates of A549 cells transfected with two control (Ctrl) or two TRAF2-targeting siRNAs and stimulated with Ago for 0.5 h were analyzed by Western blotting with antibodies against the indicated proteins. h. e. – high exposure. Vinculin was used as a loading control

Since activation of NF-κB signaling depends on the formation of a complex between the receptor and TRAF adaptors [20], we decided to examine this binding under the conditions of normal and low membrane cholesterol levels. We measured the interactions between LTβR and TRAF2 in the lysates of cells stimulated for 0.5 h with LTα1β2 in the presence or absence of MβCD. Additionally, we examined binding of TRAF2 to the receptor without stimulation. As expected, the results of immunoprecipitation assay showed no significant binding in unstimulated cells and clear interactions upon the receptor ligation (Fig. 4a). However, we were unable to find significant differences in the amounts of TRAF2 bound to LTβR between control and cholesterol-deprived cells. In the lysates of cells stimulated with LTα1β2 (under both, normal and lowered cholesterol conditions) we detected an additional form of TRAF2, with higher molecular weight. Moreover, this protein was also present in LTβR co-immunoprecipitates, where its amounts were twofold higher upon incubation with MβCD in comparison to normal condition.

As we already demonstrated, cholesterol depletion strongly enhances LTβR-dependent degradation of the pathway inhibitor IκBα. This process depends on the activity of the IKK complex, regulated by the NEMO subunit. Given evidence that exogenously expressed LTβR interacts with NEMO [63, 64] we tested if this interaction occurred between the endogenously expressed proteins and if it was sensitive to the cholesterol content of the PM. We found that stimulation of LTβR with LTα1β2 led to a modification of NEMO protein that was manifested by appearance of an additional, higher molecular weight, band on Western blot (Fig. 4b). The amounts of a modified form of NEMO further increased under low cholesterol conditions. Since activation of the NF-κB pathway by TNFα requires NEMO ubiquitylation [26, 65], we examined if the additional band on Western blot recognized by anti-NEMO antibody represented the ubiquitylated protein. Pretreatment of cells with TAK-243 [66], ubiquitylation inhibitor precluded appearance of modified form of NEMO in LTα1β2-stimulated cells under both, normal and low-cholesterol conditions (Fig. 4c). The results of co-immunoprecipitation assay showed that specifically this form of NEMO was recruited by LTβR after stimulation with the ligand (Fig. 4d). Of note, the binding between these two proteins increased in cells deprived of the PM cholesterol (Fig. 4d). Moreover, we observed that appearance of this form of NEMO was TRAF2-dependent. Silencing of TRAF2 precluded NEMO modification upon LTβR stimulation under both, normal and low-cholesterol conditions (Fig. 4e).

Altogether, these data show that stimulation of the receptor results in modifications of TRAF2 and NEMO proteins. To the best of our knowledge, LTβR interactions with the modified forms of endogenously expressed TRAF2 and NEMO were not reported before. Moreover, acute depletion of cholesterol strongly enhances these interactions.

### Hyper-activation of the NF-κB pathway in cells deprived of the PM cholesterol potentiates transcriptional response to LTβR stimulation

**Fig. 5:**
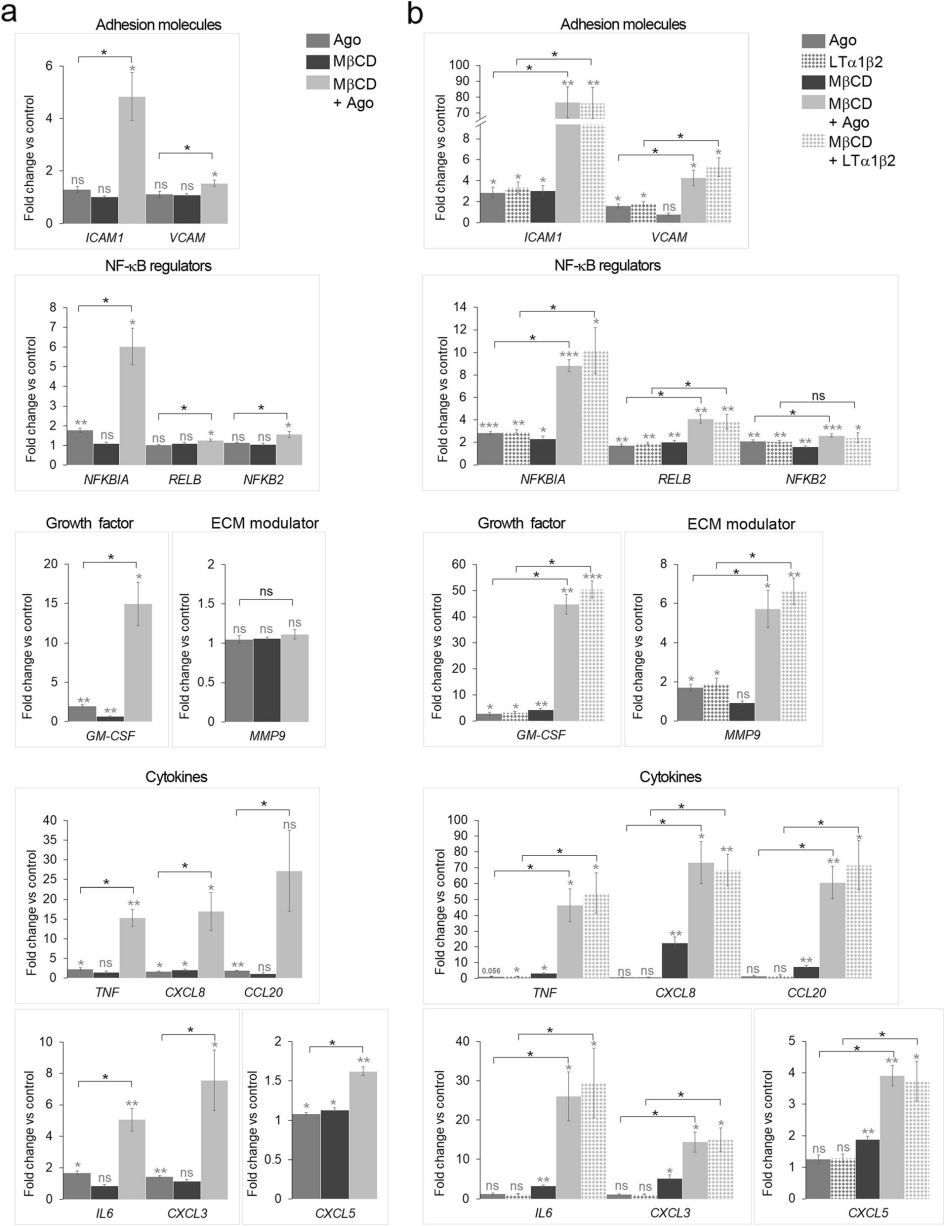
Cholesterol depletion enhances LTβR-triggered expression of NF-κB target genes. **a, b** mRNA levels of the indicated NF-κB target genes in A549 cells preincubated for 1 h with vehicle or MβCD and then stimulated for 1 h (a) or 4 h (b) with LTβR agonist (Ago) (a, b) or lymphotoxin α1β2 (LTα1β2) (b). Values are presented as fold change versus control – unstimulated and untreated cells, set as 1. Data represent the means ± SEM, *n* = 4. ns - *P* > 0.05; *P ≤ 0.05; **P ≤ 0.01; ***P ≤ 0.001 by one sample t-test (in grey) or by Mann-Whitney or Student’s t-test (in black)

As shown so far, cholesterol depletion leads to the hyper-activation of the NF-κB pathway in response to LTβR stimulation that was measured by the sustained degradation of the pathway inhibitor IκBα and enhanced phosphorylation of RelA. Next, we asked if the activation of the pathway resulted in a transcriptional response. Thus, we measured mRNA levels of the known NF-κB target genes encoding: adhesion molecules (*ICAM1*, *VCAM*), regulators of the NF-κB pathway (*NFKBIA*, *RELB*, *NFKB2*), granulocyte-macrophage colony-stimulating factor (*GM-CSF*), metalloproteinase-9 (*MMP9*), and a panel of cytokines (*TNF*, *CXCL8*, *CCL20*, *IL6*, *CXCL3*, *CXCL5*, *CCL5*) that were shown to be upregulated upon LTβR stimulation in different cell lines [11, 53, 55]. Taking into account a strong activation of NF-κB signaling in cells depleted of cholesterol upon short stimulation of LTβR, we measured the expression of the selected genes at an early time point. Stimulation with Ago for 1 h upregulated expression of several genes (*NFKBIA, GM-CSF, TNF, CXCL8, CCL20, IL6, CXCL3, CXCL5*) in control cells not treated with MβCD. The levels of these transcripts were further significantly elevated in cells co-treated with MβCD (Fig. 5a). Noteworthy, MβCD alone did not activate the expression of these genes (except for minor effects on *CXCL8* and *CXCL5*).

Consistently, we found that longer (4 h) stimulation of A549 cells with Ago or LTα1β2 upregulated transcription of most tested genes under normal conditions, as expected (Fig. 5b). Although upon 4 h incubation MβCD alone caused minor changes in transcription of some genes, it nevertheless further potentiated ligand-dependent expression of all investigates target genes (Fig. 5b). The highest increase was observed for *ICAM1*, *CXCL8* and *CCL20* genes that were upregulated 70–80 fold in comparison to unstimulated control cells or about 20–50 fold in comparison to stimulated cells under normal cholesterol conditions.

To confirm our observations, we measured the expression of selected genes in another lung cancer cell line H2228, which expresses lower levels of LTβR as compared with A549 cells (Additional file 1: Figure S6a). Upon 2 h of stimulation with Ago we observed a similar tendency toward strong upregulation of target gene expression in cholesterol-deprived cells (Additional file 1: Figure S6b).

Our findings suggest that cells with the reduced PM cholesterol level robustly respond to LTβR stimulation with increased transcription of NF-κB target genes. These data confirm synergistic effects of MβCD and LTβR ligand on NF-κB signaling.

### LTβR stimulation in cells deprived of the PM cholesterol leads to a robust pro-inflammatory response

**Fig. 6:**
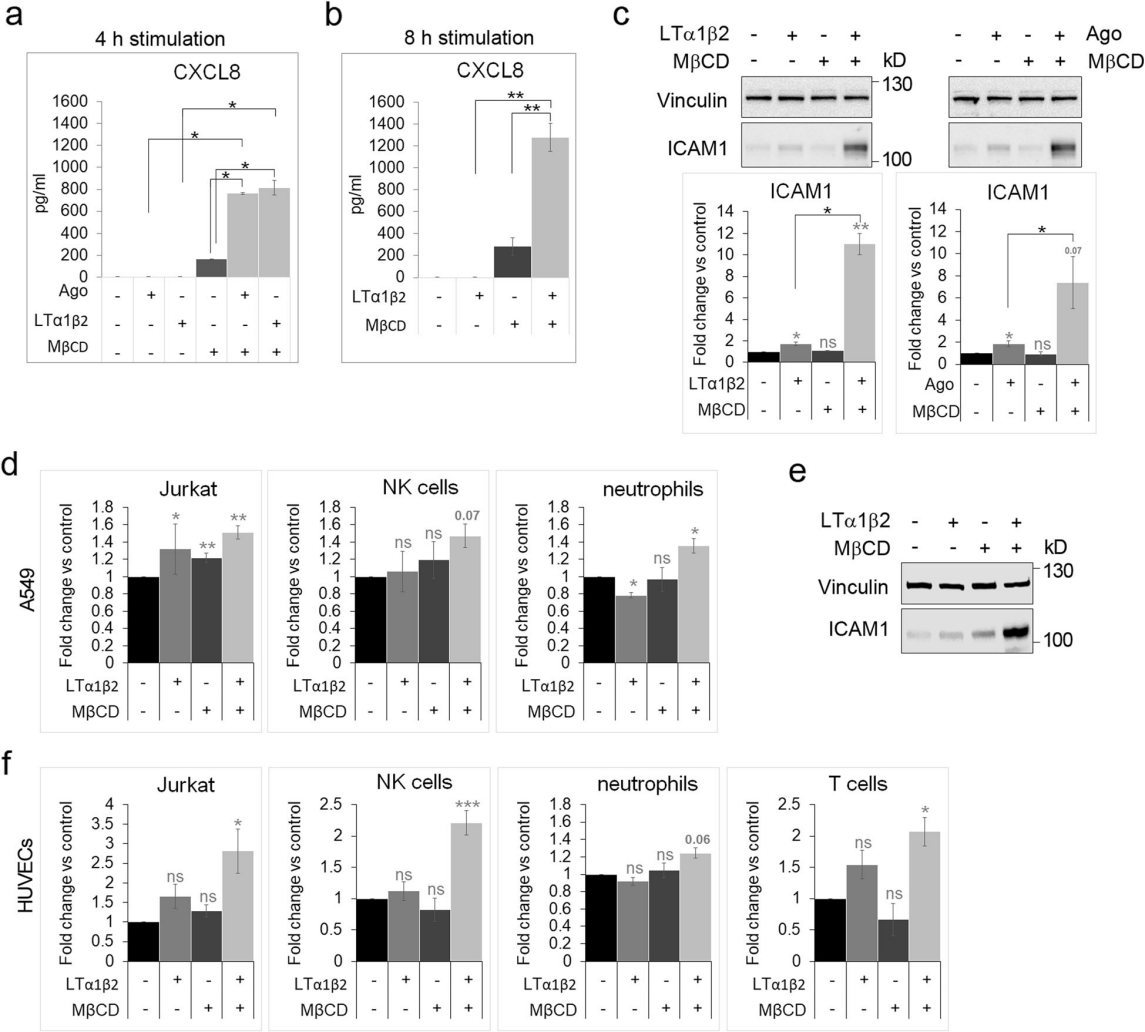
Cholesterol depletion hyper-activates LTβR-dependent pro-inflammatory response **a, b** The concentrations of secreted CXCL8 were measured with ELISA in media collected from cells preincubated for 1 h with MβCD and then stimulated or not for 4 h (a) or 8 h (b) with Ago or LTα1β2 in the presence or absence of MβCD. Data represent the means ± SEM, n = 4. *P ≤ 0.05; **P ≤ 0.01 by Mann-Whitney or Student’s t-test. **c** Lysates of A549 cells pretreated for 1 h with MβCD and then stimulated or not for 8 h with LTα1β2 or Ago in the presence or absence of MβCD were analyzed by Western blotting with antibodies against the indicated proteins. Vinculin was used as a loading control. Graph shows densitometric analysis for ICAM1 from Western blotting (protein levels normalized to vinculin). Values are presented as fold change versus controls - unstimulated and untreated cells (black bars). Data represent the means ± SEM, n = 4; ns - *P* > 0.05; *P ≤ 0.05; **P ≤ 0.01 by one sample t-test (in grey) or by Mann-Whitney (in black). **d, f** Adhesion of Jurkat, NK cells, neutrophils and T lymphocytes to A549 cells (d) and HUVECs (f) treated as in a or e, respectively. Graphs represent quantification of immune cell adhesion to A549 and HUVECs relative to control (untreated) cells. Values are presented as fold change versus controls - unstimulated and untreated cells (black bars). Data represent the means ± SEM, n = 3 (d), *n* ≥ 3 (f); ns - *P* > 0.05; *P ≤ 0.05; **P ≤ 0.01 by one sample t-test. **e** Lysates of HUVECs preincubated for 1 h with MβCD and then stimulated or not for 6 h with LTα1β2 in the presence or absence of MβCD were analyzed by Western blotting with antibodies against the indicated proteins. Vinculin was used as a loading control.

As we noticed substantial upregulation of a pro-inflammatory response at the transcriptional level, we next examined the levels of proteins encoded by the selected hyper-activated genes: CXCL8 and ICAM1 that play important roles during inflammation. CXCL8 is a chemokine involved in attraction of neutrophils and T lymphocytes [67], whereas ICAM1 is an adhesion molecule that is crucial for interactions of immune cells with endothelial and epithelial cells [68–71]. The results of ELISA assay showed that cholesterol depletion alone increased secretion of CXCL8 that was further potentiated upon stimulation with Ago or LTα1β2 for 4 and 8 h (Fig. 6a, b). Under normal cholesterol level conditions, stimulation of A549 cells with ligands did not promote the secretion of the chemokine at these time points. However, prolonged stimulation (8 h) resulted in a slight upregulation of ICAM1 protein (Fig. 6c). In cells deprived of cholesterol, administration of Ago or LTα1β2 led to a significant increase in ICAM1 levels, that was 3.5 to 5-times higher than in cells with normal cholesterol levels. Importantly, cholesterol depletion alone did not exert any effects on ICAM1 protein levels.

To test if increased production of ICAM1 was sufficient to exert a physiological effect, we measured the adherence of various types of immune cells to epithelial A549 cells upon stimulation with LTα1β2 (for 8 h) under normal and low cholesterol conditions along with control unstimulated conditions. We found the highest adhesion of Jurkat cells to A549 cells that were co-treated with MβCD and LTα1β2 (Fig. 6d).

Since ICAM1 plays a crucial role in leukocyte egress from the blood stream through the endothelial barrier, we performed experiments with HUVEC endothelial cells, which were recently shown to activate a pro-inflammatory response including ICAM1 over-production and increase of interactions with immune cells upon 24 h stimulation of LTβR [55]. We examined the adherence of Jurkat, NK cells, neutrophils and T cells to HUVECs, treated analogically to A549 cells. After 6 h of stimulation with LTα1β2 in the presence of MβCD, we found strong up-regulation of ICAM1 levels in HUVECs (Fig. 6e), that was accompanied by a significant increase of adhesion of all immune cells tested (Fig. 6f).

Cumulatively, our findings suggest that the activation of LTβR-dependent signaling upon cholesterol depletion enhances a pro-inflammatory response and promotes interactions of A549 lung cancer and endothelial cells with diverse types of immune cells.

## Discussion

### Cholesterol depletion in therapies

Since cytokine receptors are targets in the treatment of diverse human diseases [1–3], it is of high importance to get insights into molecular mechanisms regulating their signaling potential. Recently, targeting of LTβR-triggered signaling was proposed as a strategy in treatment of cancer resistant to PD-L1 blockade [17]. Tumor infiltration by T lymphocytes that enabled to overcome the resistance was enhanced by LTβR-activation resulting in overproduction of chemokines and adhesion molecules. Since targeting PD-1/PD-L1 immune checkpoint gives promising results in patients, it is of particular interest to find a way to make cancer cells more sensitive to this therapy. The data obtained in this study shed light on the relatively poorly characterized regulation of LTβR-dependent NF-κB signaling. We found that cells depleted of the PM cholesterol responded more robustly to the stimulation of the receptor with agonistic antibody or its natural ligand – lymphotoxin α1β2. This was manifested by more efficient degradation of the pathway inhibitor IκBα, increased phosphorylation of RelA, more robust transcription of NF-κB target genes and finally increased secretion of chemokine CXCL8 and expression of adhesion molecule ICAM1. The latter promotes adhesion of diverse immune cells to epithelial A549 cells and endothelial HUVECs. All these changes contribute to an inflammatory response. Our findings clearly indicate that the PM cholesterol affects the signaling outcome of LTβR. Thus hyper-activation of LTβR signaling by cholesterol depletion could potentially improve the therapeutic strategy and help to develop new ones.

MβCD treatment was already proposed as a drug in therapy of synucleinopathies since it reduced accumulation of α-synuclein in the neuronal cell body and synapses [39]. MβCD enhanced the cytotoxic effect of tamoxifen in melanoma cells [40] and induced cell death in T24 high-grade invasive urothelial cancer cells that exhibit high basal level of cholesterol. According to our data, MβCD sensitizes cells to the stimulation of LTβR. This could be of particular importance in the development of LTβR-based therapies in cases of low responsiveness to LTβR stimulation.

### Cholesterol and pro-inflammatory signaling

In our study we manipulated cholesterol in diverse ways: we sequestered it with filipin or reduced its levels in a spatially restricted manner (the PM) with MβCD or in a systematic manner by inhibiting its synthesis with simvastatin. Filipin-mediated sequestration and MβCD-mediated depletion of cholesterol in unstimulated cells did not exert any or only very small effects on the processes that we examined. Importantly, a combination of these treatments with the receptor ligation activated pro-inflammatory NF-κB signaling in a synergistic manner. Changes in the cholesterol content at the PM clearly sensitized cells for the receptor stimulation. To our surprise, we were unable to observe a synergistic effect of LTβR ligation and cholesterol depletion in cells treated with simvastatin. Moreover, we observed that inhibition of cholesterol biosynthesis activated the NF-κB pathway in a ligand-independent manner, that was in contrast to the results obtained in experiments employing filipin and MβCD. It is plausible that in simvastatin-treated cells the NF-κB pathway reached almost its full activity that could not be further increased by stimulation of the receptor. Pro-inflammatory effects of a statin reported here were already documented elsewhere [72, 73]. However, the majority of the literature data point to inhibitory effects of statins on the NF-κB pathway [74–77]. We cannot exclude that different effects of statins on pro-inflammatory signaling can be cell-type dependent. As in our experimental model simvastatin and LTβR stimulation were unable to hyper-activate the NF-κB pathway, we did not explore the potential of cholesterol synthesis inhibition in the regulation of LTβR signaling in more details.

### Cholesterol and intracellular transport of the receptor

In our studies we observed that all types of manipulation of cholesterol impaired LTβR internalization upon ligand binding. Although there are many examples of a positive role of cholesterol in the regulation of endocytosis [78–80], that support our observations, there are also contradictory data concerning the effects of statins on receptor internalization. Zhang et al. showed that lovastatin-mediated depletion of cholesterol promoted ErbB2 internalization in breast cancer cell lines [34], that is in contrast to our data on LTβR. The explanation for the discrepancy could be the fact that different receptors localize to different micro-domains at the PM and can be internalized via diverse endocytic routes. Our data indicate that LTβR internalization depends to a large extent, however not completely, on cholesterol. This conclusion is supported by the observation that impairment of caveolae-mediated endocytosis, that is largely linked to cholesterol-rich domains, reduced internalization of ligand-bound LTβR. This reduction was observed in cells depleted of cavin-1, which is involved in caveolae-dependent endocytosis [81]. The lack of effect on LTβR internalization upon caveolin-1 depletion can be explained by the presence of caveolin-2 in A549 cells, that can potentially overtake the role of caveolin-1.

The microscopic inspections of changes caused by agents targeting cholesterol revealed that two of them, filipin and MβCD, did not affect the total levels or the cellular distribution of LTβR. In contrast, inhibition of cholesterol biosynthesis by simvastatin led to intracellular accumulation of total LTβR, manifested by an increased number of LTβR-harboring vesicles as well as total amounts of the receptor. A possible explanation could be that chronic cholesterol depletion impaired secretion of LTβR, that remained sequestered in the transport vesicles on the way to the Golgi apparatus and could not reach the PM, from where under normal conditions it should be internalized and sent for degradation [50]. It was documented that cholesterol is important for the transport of secretory membrane proteins from the endoplasmic reticulum to the Golgi [82]. Thus, a lower molecular weight of the receptor accumulated upon simvastatin treatment can suggest the impairment of its transport to the Golgi compartment, where posttranslational modifications (such as glycosylation) of transmembrane proteins take place. As simvastatin alone activated NF-κB signaling in a ligand- and LTβR-independent manner, caused general deregulation of the endocytic machinery (manifested by the increased number of EEA1-positive endosomes) and likely impaired secretory transport of LTβR, we did not proceed with exploring the mechanisms of its action on LTβR in the current study.

### Mechanisms of cholesterol-dependent regulation of receptor activity

It was already demonstrated that lowering of cholesterol levels affects the activity of a diverse set of receptors. However, the underlying molecular mechanisms are poorly studied, with only a few exceptions. Pro-apoptotic effects of TRAIL acting via DR4/5 receptors depended on the recruitment of the DISC complex to the cholesterol-rich PM domains. Their disruption by MβCD resulted in the inhibition of TRAIL-induced caspase-8 cleavage and cell death [38]. The PM levels of the pro-oncogenic ErbB2 receptor were shown to be positively regulated by cholesterol [34]. Cholesterol lowering promoted internalization and lysosomal degradation of ErbB2 that decreased its PM pool. Treatment with lovastatin (inhibitor of cholesterol biosynthesis, alternative to simvastatin) improved the therapeutic potential of anti-cancer drugs targeting ErbB2, lapatinib or neratinib. Other studies showed that MβCD-mediated depletion of cholesterol sensitized cancer cells to apoptosis by an unknown mechanism [83]. Inhibition of cholesterol synthesis was also shown to change the structure of multiple-pass transmembrane proteins such as CD20 and glucose transporters [84, 85].

According to the accepted model of NF-κB signaling initiated by TNFRSF members, binding between a receptor and TRAF proteins is a prerequisite for the degradation of the pathway inhibitor IκBα that is indirectly dependent on the IKK complex. Here, we provide evidence that upon stimulation with a ligand, LTβR recruits a ubiquitylated NEMO, the IKK complex subunit. So far recruitment of NEMO to a transmembrane receptor was documented for the founder of TNFRSF family - TNFR1 [86–88], epidermal growth factor receptor (EGFR) [89], TYRO3, TEK and other receptor tyrosine kinases [90]. However, most of these data including the results concerning interactions of LTβR and NEMO [63, 64] come from experiments employing ectopically expressed proteins. Additionally, we showed that the uncovered binding between endogenous LTβR and modified NEMO was significantly enhanced under the low cholesterol content conditions, presumably due to increased amounts of ubiquitylated NEMO. It was shown that cholesterol depletion could promote ubiquitylation of NPC1 protein [91]. At the same time, complexes of the receptor with a modified form of TRAF2 were also more abundant under low cholesterol- in comparison to normal cholesterol level conditions. It is possible that increased binding between LTβR and the modified forms of NEMO and TRAF2 may contribute to the observed efficient degradation of the pathway inhibitor resulting in enhanced activation of NF-κB signaling. Nevertheless, the spatial and temporal details of the events following ligand binding to the receptor at the PM depleted of cholesterol remain unknown. One may speculate that upon cholesterol depletion the proportion of the receptor anchored in non-lipid rafts increases, and is more prone to recruit TRAF and NEMO proteins that activate the NF-κB pathway. This idea is supported by a previous study showing that NEMO associates preferentially with non-lipid raft fractions of the PM and furthermore the integrity of lipid rafts is dispensable for the activation of NF-κB by TNFR1 [36]. A similar mechanism could operate for LTβR but its molecular details await further investigation.

## Supplementary information

**Additional file 1: Figure S1.** LTβR stimulation in A549 cells leads to activation of the canonical NF-κB pathway. **a, b** Lysates of A549 cells stimulated for the indicated times with LTβR agonist (Ago) **(a)** or lymphotoxin α1β2 (LTα1β2) **(b)** were analyzed by Western blotting with antibodies against the indicated proteins. Vinculin was used as a loading control. Graphs show densitometric analysis for P-RelA and IκBα from Western blotting (protein levels normalized to vinculin). Values are presented as fold change versus control - unstimulated cells (black bars). Data represent the means ± SEM, *n* ≥ 5; ns - *P* > 0.05; *P ≤ 0.05; **P ≤ 0.01; ***P ≤ 0.001 by one sample t-test. **Figure S2.** Inhibition of cholesterol synthesis by simvastatin activates NF-κB signaling and affects internalization of ligand-bound LTβR and its total cellular levels. **a** Lysates of A549 cells preincubated for 48 h in delipidated medium containing simvastatin or vehicle and stimulated for 0.5 or 1 h with LTβR agonist (Ago) were analyzed by Western blotting with antibodies against the indicated proteins. Vinculin was used as a loading control. Graphs show densitometric analysis of the indicated proteins from Western blotting (protein levels normalized to vinculin). Values are presented as fold change versus controls - unstimulated and untreated cells (black bars). Data represent the means ± SEM, *n* = 3; ns - *P* > 0.05; **P ≤ 0.01 by one sample t-test (in grey) or Student’s t-test (in black). **b** Immunofluorescence staining of ligand-bound LTβR and EEA1 in A549 cells upon 0.5 h stimulation with LTβR agonist in medium containing vehicle (Veh.) or simvastatin. Insets: magnified views of boxed regions in the main images. Scale bars, 20 μm. Graphs: analysis of integral intensity and number of LTβR- and EEA1-positive vesicles in cells treated as in B. Values are presented as fold change versus controls - vehicle-treated cells marked as a black line, set as 1. Data represent the means ± SEM, *n* = 3. ns - *P* > 0.05; *P ≤ 0.05; **P ≤ 0.01 by one sample t-test. **c** Immunofluorescence staining of total LTβR and EEA1 in A549 cells treated for 48 h with either vehicle (Veh.) or simvastatin. Insets: Magnified views of boxed regions in the main images. Scale bars, 20 μm. Graphs: analysis of integral intensity and number of LTβR- and EEA1-positive vesicles in cells treated as in C. Values are presented as fold change versus controls - vehicle-treated cells marked as a black line, set as 1. Data represent the means ± SEM, *n* = 3. ns - *P* > 0.05; *P ≤ 0.05 by one sample t-test. **d** Lysates of A549 cells stimulated for 0.5 h with LTβR agonist (Ago) preincubated for 48 h with simvastatin or vehicle were analyzed by Western blotting with antibodies against the indicated proteins. Vinculin was used as a loading control. Graph show densitometric analysis of LTβR from Western blotting (protein levels normalized to vinculin). Values are presented as fold change versus controls – unstimulated and untreated cells (black bar). Data represent the means ± SEM, n = 3; ns - *P* > 0.05; **P ≤ 0.01 by one sample t-test. **(e)** LTβR mRNA level in cells incubated with simvastatin. Values are presented as fold change versus controls – vehicle-treated cells (black bar). Data represent the means ± SEM, n = 3 ns - *P* > 0.05 by one sample t-test. **Figure S3.** Inhibition of cholesterol synthesis by simvastatin activates NF-κB signaling in LTβR-independent manner. A549 cells edited with the CRISPR/Cas9 method with two LTβR-targeting sgRNAs and two non-targeting (NT) sgRNAs were incubated with simvastatin or vehicle for 48 h. Cell lysates were analyzed by Western blotting with antibodies against the indicated proteins. Vinculin was used as a loading control. Graphs show densitometric analysis of the indicated proteins from Western blotting (protein levels normalized to vinculin). Values are presented as fold change versus controls - untreated cells (black bars). Data represent the means ± SEM, n = 3; ns - *P* > 0.05; *P ≤ 0.05 by one sample t-test. **Figure S4.** Cholesterol depletion by MβCD does not influence LTβR distribution within a cell. **a** Immunofluorescence staining of total LTβR and EEA1 in A549 cells upon 1 h incubation with MβCD or vehicle (Veh.). Insets: magnified views of boxed regions in the main images. Scale bars, 20 μm. **b** Analysis of integral intensity and the number of LTβR- and EEA1-positive vesicles in cells treated as in A. Values are presented as fold change versus control - vehicle-treated cells marked as a black line, set as 1. Data represent the means ± SEM, *n* = 3. ns - *P* > 0.05 by one sample t-test. **Figure S5**. Perturbation in caveolae-dependent endocytosis does not affect LTβR-dependent signaling. **a** Immunofluorescence staining of ligand-bound LTβR, EEA1 and caveolin-1 in A549 cells upon knockdown of caveolin-1 or in control (Ctrl) siRNA-transfected cells upon 0.5 h stimulation with Ago. Two siRNAs targeting caveolin-1 and two non-targeting siRNAs were used. Insets: magnified views of boxed regions in the main images. Scale bars, 20 μm. Graphs present analysis of integral intensity and the number of LTβR- and EEA1-positive vesicles in cells treated as in A. Values are presented as fold change versus cells transfected with non-targeting siRNA (Ctrl_1) marked as a black line, set as 1. Data represent the means ± SEM, n = 3. ns - *P* > 0.05; *P ≤ 0.05; **P ≤ 0.01 by one sample t-test. **b** Immunofluorescence staining of ligand-bound LTβR and EEA1 in A549 cells upon knockdown of cavin-1 or in control (Ctrl) siRNA-transfected cells upon 0.5 h stimulation with Ago. Two siRNAs targeting cavin-1 and two non-targeting siRNAs were used. Insets: magnified views of boxed regions in the main images. Scale bars, 20 μm. Graphs present analysis of integral intensity and number of LTβR- and EEA1-positive vesicles in cells treated as in A. Values are presented as fold change versus cells transfected with non-targeting siRNA (Ctrl_1) marked as a black line, set as 1. Data represent the means ± SEM, *n* = 5. ns - *P* > 0.05; *P ≤ 0.05; **P ≤ 0.01; ***P ≤ 0.001 by one sample t-test. **c** Lysates of A549 cells transfected with control (Ctrl) or cavin-1 targeting siRNAs (two oligonucleotides per gene) and stimulated with Ago for 0.5 and 1 h were analyzed by Western blotting with antibodies against the indicated proteins. Vinculin was used as a loading control. Graph shows densitometric analysis of IκBα from Western blotting (protein levels normalized to vinculin). Values are presented as fold change versus controls - unstimulated and untreated cells (black bars). Data represent the means ± SEM, *n* = 4; ns - *P* > 0.05; *P ≤ 0.05 by one sample t-test. **Figure S6.** Cholesterol depletion enhances LTβR-triggered expression of NF-κB target genes in H2228 cell line. **a** Lysates of H2228 cells were analyzed by Western blotting with antibodies against the indicated proteins. Vinculin was used as a loading control. **b** mRNA levels of the indicated NF-κB target genes in H2228 cells pretreated for 1 h with vehicle or MβCD and then stimulated for 2 h with LTβR agonist (Ago). Values are presented as fold change versus control – unstimulated and untreated cells, set as 1. Data represent the means ± SEM, *n* = 4. ns - *P* > 0.05; *P ≤ 0.05; **P ≤ 0.01; ***P ≤ 0.001 by one sample t-test (in grey) or by Mann-Whitney or Student’s t-test (in black).

**Additional file 2: Table S1.** Antibodies used for Western blotting. **Table S2.** Antibodies used for Immunofluorescence. **Table 3.** List of sequences of sgRNA used. **Table 4.** Sequences of primers used for qRT-PCR. **Table 5.** TaqMan® Gene Expression Assays used for qRT-PCR

## Data Availability

The datasets used and/or analyzed during the current study are available on reasonable request.

## Abbreviations

Ago: Agonistic anti-LTβR antibody
AP-1: Activator protein 1
CXCL8: Chemokine (C-X-C motif) ligand 8
DR: Death receptor
EEA1: Early endosome antigen 1
ERK1: Extracellular signal–regulated kinase 1
ICAM1: Intercellular adhesion molecule 1
LIGHT: Homologous to lymphotoxin, exhibits inducible expression and competes with HSV glycoprotein D for binding to herpesvirus entry mediator, a receptor expressed on T lymphocytes
LT α1β2: Lymphotoxin α1β2
LTβR: Lymphotoxin β receptor
MβCD: Methyl-β-cyclodextrin
NEMO: NF-κB essential modulator
NF-κB: Nuclear factor kappa-light-chain-enhancer of activated B cells
NIK: NF-κB-inducing kinase
NK: Natural killer
NSCLC: Non-small-cell lung carcinoma
PM: Plasma membrane
TNFRSF: Tumor necrosis factor receptor superfamily
TRAF: Tumor necrosis factor receptor associated factor
TRAIL: TNF-related apoptosis-inducing ligand

## References

[1] Browning JL (2008). Inhibition of the lymphotoxin pathway as a therapy for autoimmune disease. Immunol Rev.

[2] Wajant H, Gerspach J, Pfizenmaier K (2005). Tumor therapeutics by design: targeting and activation of death receptors. Cytokine Growth Factor Rev.

[3] Tamada K, Chen L (2006). Renewed interest in cancer immunotherapy with the tumor necrosis factor superfamily molecules. Cancer immunology, immunotherapy : CII.

[4] Crowe PD, VanArsdale TL, Walter BN, Ware CF, Hession C, Ehrenfels B (1994). A lymphotoxin-beta-specific receptor. Science..

[5] Mauri DN, Ebner R, Montgomery RI, Kochel KD, Cheung TC, Yu GL (1998). LIGHT, a new member of the TNF superfamily, and lymphotoxin alpha are ligands for herpesvirus entry mediator. Immunity..

[6] Yilmaz ZB, Weih DS, Sivakumar V, Weih F (2003). RelB is required for Peyer's patch development: differential regulation of p52-RelB by lymphotoxin and TNF. EMBO J.

[7] Futterer A, Mink K, Luz A, Kosco-Vilbois MH, Pfeffer K (1998). The lymphotoxin beta receptor controls organogenesis and affinity maturation in peripheral lymphoid tissues. Immunity..

[8] Iizuka K, Chaplin DD, Wang Y, Wu Q, Pegg LE, Yokoyama WM (1999). Requirement for membrane lymphotoxin in natural killer cell development. Proc Natl Acad Sci U S A.

[9] Smyth MJ, Johnstone RW, Cretney E, Haynes NM, Sedgwick JD, Korner H (1999). Multiple deficiencies underlie NK cell inactivity in lymphotoxin-alpha gene-targeted mice. J Immunol.

[10] Fu YX, Huang G, Matsumoto M, Molina H, Chaplin DD (1997). Independent signals regulate development of primary and secondary follicle structure in spleen and mesenteric lymph node. Proc Natl Acad Sci U S A.

[11] Piao W, Xiong Y, Famulski K, Brinkman CC, Li L, Toney N (2018). Regulation of T cell afferent lymphatic migration by targeting LTbetaR-mediated non-classical NFkappaB signaling. Nat Commun.

[12] Seleznik G, Seeger H, Bauer J, Fu K, Czerkowicz J, Papandile A (2016). The lymphotoxin beta receptor is a potential therapeutic target in renal inflammation. Kidney Int.

[13] Fava RA, Kennedy SM, Wood SG, Bolstad AI, Bienkowska J, Papandile A (2011). Lymphotoxin-beta receptor blockade reduces CXCL13 in lacrimal glands and improves corneal integrity in the NOD model of Sjogren's syndrome. Arthritis research & therapy.

[14] Gatumu MK, Skarstein K, Papandile A, Browning JL, Fava RA, Bolstad AI (2009). Blockade of lymphotoxin-beta receptor signaling reduces aspects of Sjogren's syndrome in salivary glands of non-obese diabetic mice. Arthritis research & therapy..

[15] Gommerman JL, Browning JL (2003). Lymphotoxin/light, lymphoid microenvironments and autoimmune disease. Nat Rev Immunol.

[16] Lukashev M, LePage D, Wilson C, Bailly V, Garber E, Lukashin A (2006). Targeting the lymphotoxin-beta receptor with agonist antibodies as a potential cancer therapy. Cancer Res.

[17] Tang H, Wang Y, Chlewicki LK, Zhang Y, Guo J, Liang W (2016). Facilitating T cell infiltration in tumor microenvironment overcomes resistance to PD-L1 blockade. Cancer Cell.

[18] Dejardin E, Droin NM, Delhase M, Haas E, Cao Y, Makris C (2002). The lymphotoxin-beta receptor induces different patterns of gene expression via two NF-kappaB pathways. Immunity..

[19] Chang YH, Hsieh SL, Chen MC, Lin WW (2002). Lymphotoxin beta receptor induces interleukin 8 gene expression via NF-kappaB and AP-1 activation. Exp Cell Res.

[20] Kuai J, Nickbarg E, Wooters J, Qiu Y, Wang J, Lin LL (2003). Endogenous association of TRAF2, TRAF3, cIAP1, and Smac with lymphotoxin beta receptor reveals a novel mechanism of apoptosis. J Biol Chem.

[21] Rooney IA, Butrovich KD, Glass AA, Borboroglu S, Benedict CA, Whitbeck JC (2000). The lymphotoxin-beta receptor is necessary and sufficient for LIGHT-mediated apoptosis of tumor cells. J Biol Chem.

[22] VanArsdale TL, VanArsdale SL, Force WR, Walter BN, Mosialos G, Kieff E (1997). Lymphotoxin-beta receptor signaling complex: role of tumor necrosis factor receptor-associated factor 3 recruitment in cell death and activation of nuclear factor kappaB. Proc Natl Acad Sci U S A.

[23] Devin A, Cook A, Lin Y, Rodriguez Y, Kelliher M, Liu Z (2000). The distinct roles of TRAF2 and RIP in IKK activation by TNF-R1: TRAF2 recruits IKK to TNF-R1 while RIP mediates IKK activation. Immunity..

[24] Vallabhapurapu S, Karin M (2009). Regulation and function of NF-kappaB transcription factors in the immune system. Annu Rev Immunol.

[25] Delhase M, Hayakawa M, Chen Y, Karin M (1999). Positive and negative regulation of IkappaB kinase activity through IKKbeta subunit phosphorylation. Science..

[26] Iwai K, Tokunaga F (2009). Linear polyubiquitination: a new regulator of NF-kappaB activation. EMBO Rep.

[27] Natoli G, Saccani S, Bosisio D, Marazzi I (2005). Interactions of NF-kappaB with chromatin: the art of being at the right place at the right time. Nat Immunol.

[28] Dejardin E (2006). The alternative NF-kappaB pathway from biochemistry to biology: pitfalls and promises for future drug development. Biochem Pharmacol.

[29] Senftleben U, Cao Y, Xiao G, Greten FR, Krahn G, Bonizzi G (2001). Activation by IKKalpha of a second, evolutionary conserved, NF-kappa B signaling pathway. Science..

[30] Xiao G, Harhaj EW, Sun SC (2001). NF-kappaB-inducing kinase regulates the processing of NF-kappaB2 p100. Mol Cell.

[31] Chen X, Resh MD (2002). Cholesterol depletion from the plasma membrane triggers ligand-independent activation of the epidermal growth factor receptor. J Biol Chem.

[32] Fukui K, Ferris HA, Kahn CR (2015). Effect of cholesterol reduction on receptor signaling in neurons. J Biol Chem.

[33] Simons K, Toomre D (2000). Lipid rafts and signal transduction. Nat Rev Mol Cell Biol.

[34] Zhang J, Li Q, Wu Y, Wang D, Xu L, Zhang Y (2019). Cholesterol content in cell membrane maintains surface levels of ErbB2 and confers a therapeutic vulnerability in ErbB2-positive breast cancer. Cell communication and signaling : CCS.

[35] Legler DF, Micheau O, Doucey MA, Tschopp J, Bron C (2003). Recruitment of TNF receptor 1 to lipid rafts is essential for TNFalpha-mediated NF-kappaB activation. Immunity..

[36] Doan JE, Windmiller DA, Riches DW (2004). Differential regulation of TNF-R1 signaling: lipid raft dependency of p42mapk/erk2 activation, but not NF-kappaB activation. J Immunol.

[37] Lewis Andrew K., Valley Christopher C., Peery Stephen L., Brummel Benjamin, Braun Anthony R., Karim Christine B., Sachs Jonathan N. (2016). Death Receptor 5 Networks Require Membrane Cholesterol for Proper Structure and Function. Journal of Molecular Biology.

[38] Song JH, Tse MC, Bellail A, Phuphanich S, Khuri F, Kneteman NM (2007). Lipid rafts and nonrafts mediate tumor necrosis factor related apoptosis-inducing ligand induced apoptotic and nonapoptotic signals in non small cell lung carcinoma cells. Cancer Res.

[39] Bar-On P, Rockenstein E, Adame A, Ho G, Hashimoto M, Masliah E (2006). Effects of the cholesterol-lowering compound methyl-beta-cyclodextrin in models of alpha-synucleinopathy. J Neurochem.

[40] Mohammad N, Malvi P, Meena AS, Singh SV, Chaube B, Vannuruswamy G (2014). Cholesterol depletion by methyl-beta-cyclodextrin augments tamoxifen induced cell death by enhancing its uptake in melanoma. Mol Cancer.

[41] Resnik N, Repnik U, Kreft ME, Sepcic K, Macek P, Turk B (2015). Highly selective anti-Cancer activity of cholesterol-interacting agents methyl-beta-Cyclodextrin and Ostreolysin a/Pleurotolysin B protein complex on Urothelial Cancer cells. PLoS One.

[42] Coisne C, Hallier-Vanuxeem D, Boucau MC, Hachani J, Tilloy S, Bricout H, et al. beta-Cyclodextrins Decrease Cholesterol Release and ABC-Associated Transporter Expression in Smooth Muscle Cells and Aortic Endothelial Cells. Frontiers in physiology. 2016;7:185. Epub 2016/06/03. 10.3389/fphys.2016.00185 PMC4879322 27252658

[43] Schneider CA, Rasband WS, Eliceiri KW (2012). NIH image to ImageJ: 25 years of image analysis. Nat Methods.

[44] Mamińska Agnieszka, Bartosik Anna, Banach-Orłowska Magdalena, Pilecka Iwona, Jastrzębski Kamil, Zdżalik-Bielecka Daria, Castanon Irinka, Poulain Morgane, Neyen Claudine, Wolińska-Nizioł Lidia, Toruń Anna, Szymańska Ewelina, Kowalczyk Agata, Piwocka Katarzyna, Simonsen Anne, Stenmark Harald, Fürthauer Maximilian, González-Gaitán Marcos, Miaczynska Marta (2016). ESCRT proteins restrict constitutive NF-κB signaling by trafficking cytokine receptors. Science Signaling.

[45] Sadowski L, Jastrzebski K, Kalaidzidis Y, Heldin CH, Hellberg C, Miaczynska M (2013). Dynamin inhibitors impair endocytosis and mitogenic signaling of PDGF. Traffic..

[46] Jastrzebski K, Zdzalik-Bielecka D, Maminska A, Kalaidzidis Y, Hellberg C, Miaczynska M (2017). Multiple routes of endocytic internalization of PDGFRbeta contribute to PDGF-induced STAT3 signaling. J Cell Sci.

[47] Collinet C, Stoter M, Bradshaw CR, Samusik N, Rink JC, Kenski D (2010). Systems survey of endocytosis by multiparametric image analysis. Nature..

[48] Kalaidzidis Y, Kalaidzidis I, Zerial M (1641). A probabilistic method to quantify the Colocalization of markers on intracellular vesicular structures visualized by light microscopy. Aip Conf Proc.

[49] Rink J, Ghigo E, Kalaidzidis Y, Zerial M (2005). Rab conversion as a mechanism of progression from early to late endosomes. Cell..

[50] Banach-Orłowska Magdalena, Jastrzębski Kamil, Cendrowski Jarosław, Maksymowicz Małgorzata, Wojciechowska Karolina, Korostyński Michał, Moreau Dimitri, Gruenberg Jean, Miaczynska Marta (2018). The topology of the lymphotoxin β receptor that accumulates upon endolysosomal dysfunction dictates the NF-κB signaling outcome. Journal of Cell Science.

[51] Doench JG, Fusi N, Sullender M, Hegde M, Vaimberg EW, Donovan KF (2016). Optimized sgRNA design to maximize activity and minimize off-target effects of CRISPR-Cas9. Nat Biotechnol.

[52] Joung J, Konermann S, Gootenberg JS, Abudayyeh OO, Platt RJ, Brigham MD (2017). Genome-scale CRISPR-Cas9 knockout and transcriptional activation screening. Nat Protoc.

[53] da Silva AR, Madge L, Soroosh P, Tocker J, Croft M (2015). The TNF family molecules LIGHT and Lymphotoxin alphabeta induce a distinct steroid-resistant inflammatory phenotype in human lung epithelial cells. J Immunol.

[54] Ganeff C, Remouchamps C, Boutaffala L, Benezech C, Galopin G, Vandepaer S (2011). Induction of the alternative NF-kappaB pathway by lymphotoxin alphabeta (LTalphabeta) relies on internalization of LTbeta receptor. Mol Cell Biol.

[55] Kucharzewska Paulina, Maracle Chrissta X., Jeucken Kim C. M., van Hamburg Jan Piet, Israelsson Elisabeth, Furber Mark, Tas Sander W., Olsson Henric K. (2019). NIK–IKK complex interaction controls NF-κB-dependent inflammatory activation of endothelium in response to LTβR ligation. Journal of Cell Science.

[56] McGookey DJ, Fagerberg K, Anderson RG (1983). Filipin-cholesterol complexes form in uncoated vesicle membrane derived from coated vesicles during receptor-mediated endocytosis of low density lipoprotein. J Cell Biol.

[57] Awasthi-Kalia M, Schnetkamp PP, Deans JP (2001). Differential effects of filipin and methyl-beta-cyclodextrin on B cell receptor signaling. Biochem Biophys Res Commun.

[58] Schnitzer JE, Oh P, Pinney E, Allard J (1994). Filipin-sensitive caveolae-mediated transport in endothelium: reduced transcytosis, scavenger endocytosis, and capillary permeability of select macromolecules. J Cell Biol.

[59] Orlandi PA, Fishman PH (1998). Filipin-dependent inhibition of cholera toxin: evidence for toxin internalization and activation through caveolae-like domains. J Cell Biol.

[60] Alberts AW (1990). Lovastatin and simvastatin--inhibitors of HMG CoA reductase and cholesterol biosynthesis. Cardiology..

[61] Harder T, Simons K (1997). Caveolae, DIGs, and the dynamics of sphingolipid-cholesterol microdomains. Curr Opin Cell Biol.

[62] Kiss AL, Botos E (2009). Endocytosis via caveolae: alternative pathway with distinct cellular compartments to avoid lysosomal degradation?. J Cell Mol Med.

[63] Huttlin EL, Ting L, Bruckner RJ, Gebreab F, Gygi MP, Szpyt J (2015). The BioPlex network: a systematic exploration of the human Interactome. Cell..

[64] Huttlin EL, Bruckner RJ, Paulo JA, Cannon JR, Ting L, Baltier K (2017). Architecture of the human interactome defines protein communities and disease networks. Nature..

[65] Tokunaga F, Sakata S, Saeki Y, Satomi Y, Kirisako T, Kamei K (2009). Involvement of linear polyubiquitylation of NEMO in NF-kappaB activation. Nat Cell Biol.

[66] Hyer ML, Milhollen MA, Ciavarri J, Fleming P, Traore T, Sappal D (2018). A small-molecule inhibitor of the ubiquitin activating enzyme for cancer treatment. Nat Med.

[67] Petering H, Gotze O, Kimmig D, Smolarski R, Kapp A, Elsner J (1999). The biologic role of interleukin-8: functional analysis and expression of CXCR1 and CXCR2 on human eosinophils. Blood..

[68] Dustin ML, Springer TA (1988). Lymphocyte function-associated antigen-1 (LFA-1) interaction with intercellular adhesion molecule-1 (ICAM-1) is one of at least three mechanisms for lymphocyte adhesion to cultured endothelial cells. J Cell Biol.

[69] Shieh CC, Sadasivan BK, Russell GJ, Schon MP, Parker CM, Brenner MB (1999). Lymphocyte adhesion to epithelia and endothelia mediated by the lymphocyte endothelial-epithelial cell adhesion molecule glycoprotein. J Immunol.

[70] Yang L, Froio RM, Sciuto TE, Dvorak AM, Alon R, Luscinskas FW (2005). ICAM-1 regulates neutrophil adhesion and transcellular migration of TNF-alpha-activated vascular endothelium under flow. Blood..

[71] Rahman A, Fazal F (2009). Hug tightly and say goodbye: role of endothelial ICAM-1 in leukocyte transmigration. Antioxid Redox Signal.

[72] Riganti C, Doublier S, Costamagna C, Aldieri E, Pescarmona G, Ghigo D (2008). Activation of nuclear factor-kappa B pathway by simvastatin and RhoA silencing increases doxorubicin cytotoxicity in human colon cancer HT29 cells. Mol Pharmacol.

[73] Nezic L, Skrbic R, Amidzic L, Gajanin R, Kuca K, Jacevic V (2018). Simvastatin protects Cardiomyocytes against endotoxin-induced apoptosis and up-regulates Survivin/NF-kappaB/p65 expression. Sci Rep.

[74] Lee JY, Kim JS, Kim JM, Kim N, Jung HC, Song IS (2007). Simvastatin inhibits NF-kappaB signaling in intestinal epithelial cells and ameliorates acute murine colitis. Int Immunopharmacol.

[75] Holschermann H, Schuster D, Parviz B, Haberbosch W, Tillmanns H, Muth H (2006). Statins prevent NF-kappaB transactivation independently of the IKK-pathway in human endothelial cells. Atherosclerosis..

[76] Ahn KS, Sethi G, Aggarwal BB (2007). Simvastatin potentiates TNF-alpha-induced apoptosis through the down-regulation of NF-kappaB-dependent antiapoptotic gene products: role of IkappaBalpha kinase and TGF-beta-activated kinase-1. J Immunol.

[77] Hilgendorff A, Muth H, Parviz B, Staubitz A, Haberbosch W, Tillmanns H (2003). Statins differ in their ability to block NF-kappaB activation in human blood monocytes. Int J Clin Pharmacol Ther.

[78] Imelli N, Meier O, Boucke K, Hemmi S, Greber UF (2004). Cholesterol is required for endocytosis and endosomal escape of adenovirus type 2. J Virol.

[79] Urs NM, Jones KT, Salo PD, Severin JE, Trejo J, Radhakrishna H (2005). A requirement for membrane cholesterol in the beta-arrestin- and clathrin-dependent endocytosis of LPA1 lysophosphatidic acid receptors. J Cell Sci.

[80] Yue HY, Xu J (2015). Cholesterol regulates multiple forms of vesicle endocytosis at a mammalian central synapse. J Neurochem.

[81] Hill MM, Bastiani M, Luetterforst R, Kirkham M, Kirkham A, Nixon SJ (2008). PTRF-Cavin, a conserved cytoplasmic protein required for caveola formation and function. Cell..

[82] Ridsdale A, Denis M, Gougeon PY, Ngsee JK, Presley JF, Zha X (2006). Cholesterol is required for efficient endoplasmic reticulum-to-Golgi transport of secretory membrane proteins. Mol Biol Cell.

[83] Yamaguchi Ryuji, Perkins Guy, Hirota Kiichi (2015). Targeting cholesterol with β-cyclodextrin sensitizes cancer cells for apoptosis. FEBS Letters.

[84] Malenda A, Skrobanska A, Issat T, Winiarska M, Bil J, Oleszczak B (2012). Statins impair glucose uptake in tumor cells. Neoplasia..

[85] Winiarska M, Bil J, Wilczek E, Wilczynski GM, Lekka M, Engelberts PJ (2008). Statins impair antitumor effects of rituximab by inducing conformational changes of CD20. PLoS Med.

[86] Nakazawa S, Oikawa D, Ishii R, Ayaki T, Takahashi H, Takeda H (2016). Linear ubiquitination is involved in the pathogenesis of optineurin-associated amyotrophic lateral sclerosis. Nat Commun.

[87] Workman LM, Habelhah H (2013). TNFR1 signaling kinetics: spatiotemporal control of three phases of IKK activation by posttranslational modification. Cell Signal.

[88] Zhang SQ, Kovalenko A, Cantarella G, Wallach D (2000). Recruitment of the IKK signalosome to the p55 TNF receptor: RIP and A20 bind to NEMO (IKKgamma) upon receptor stimulation. Immunity..

[89] Petschnigg J, Groisman B, Kotlyar M, Taipale M, Zheng Y, Kurat CF (2014). The mammalian-membrane two-hybrid assay (MaMTH) for probing membrane-protein interactions in human cells. Nat Methods.

[90] Fenner BJ, Scannell M, Prehn JH (2010). Expanding the substantial interactome of NEMO using protein microarrays. PLoS One.

[91] Ohsaki Y, Sugimoto Y, Suzuki M, Hosokawa H, Yoshimori T, Davies JP (2006). Cholesterol depletion facilitates ubiquitylation of NPC1 and its association with SKD1/Vps4. J Cell Sci.

