# Supplementary material for: Cholesterol restricts lymphotoxin β receptor-triggered NF-κB signaling
Source: Cell Commun Signal. 2019 Dec 26;17:171. doi: 10.1186/s12964-019-0460-1 (PMC6933913; doi:10.1186/s12964-019-0460-1)
Supplement: Supplementary file 2 — Additional file 1: Figure S1. LTβR stimulation in A549 cells leads to activation of the canonical NF-κB pathway. a, b Lysates of A549 cells stimulated for the indicated times with LTβR agonist (Ago) (a) or lymphotoxin α1β2 (LTα1β2) (b) were analyzed by Western blotting with antibodies against the indicated proteins. Vinculin was used as a loading control. Graphs show densitometric analysis for P-RelA and IκBα from Western blotting (protein levels normalized to vinculin). Values are presented as fold change versus control - unstimulated cells (black bars). Data represent the means ± SEM, n ≥ 5; ns - P > 0.05; *P ≤ 0.05; **P ≤ 0.01; ***P ≤ 0.001 by one sample t-test. Figure S2. Inhibition of cholesterol synthesis by simvastatin activates NF-κB signaling and affects internalization of ligand-bound LTβR and its total cellular levels. a Lysates of A549 cells preincubated for 48 h in delipidated medium containing simvastatin or vehicle and stimulated for 0.5 or 1 h with LTβR agonist (Ago) were analyzed by Western blotting with antibodies against the indicated proteins. Vinculin was used as a loading control. Graphs show densitometric analysis of the indicated proteins from Western blotting (protein levels normalized to vinculin). Values are presented as fold change versus controls - unstimulated and untreated cells (black bars). Data represent the means ± SEM, n = 3; ns - P > 0.05; **P ≤ 0.01 by one sample t-test (in grey) or Student’s t-test (in black). b Immunofluorescence staining of ligand-bound LTβR and EEA1 in A549 cells upon 0.5 h stimulation with LTβR agonist in medium containing vehicle (Veh.) or simvastatin. Insets: magnified views of boxed regions in the main images. Scale bars, 20 μm. Graphs: analysis of integral intensity and number of LTβR- and EEA1-positive vesicles in cells treated as in B. Values are presented as fold change versus controls - vehicle-treated cells marked as a black line, set as 1. Data represent the means ± SEM, n = 3. ns - P > 0.05; *P ≤ [file 12964_2019_460_MOESM2_ESM.docx]

**Additional file 1**

**
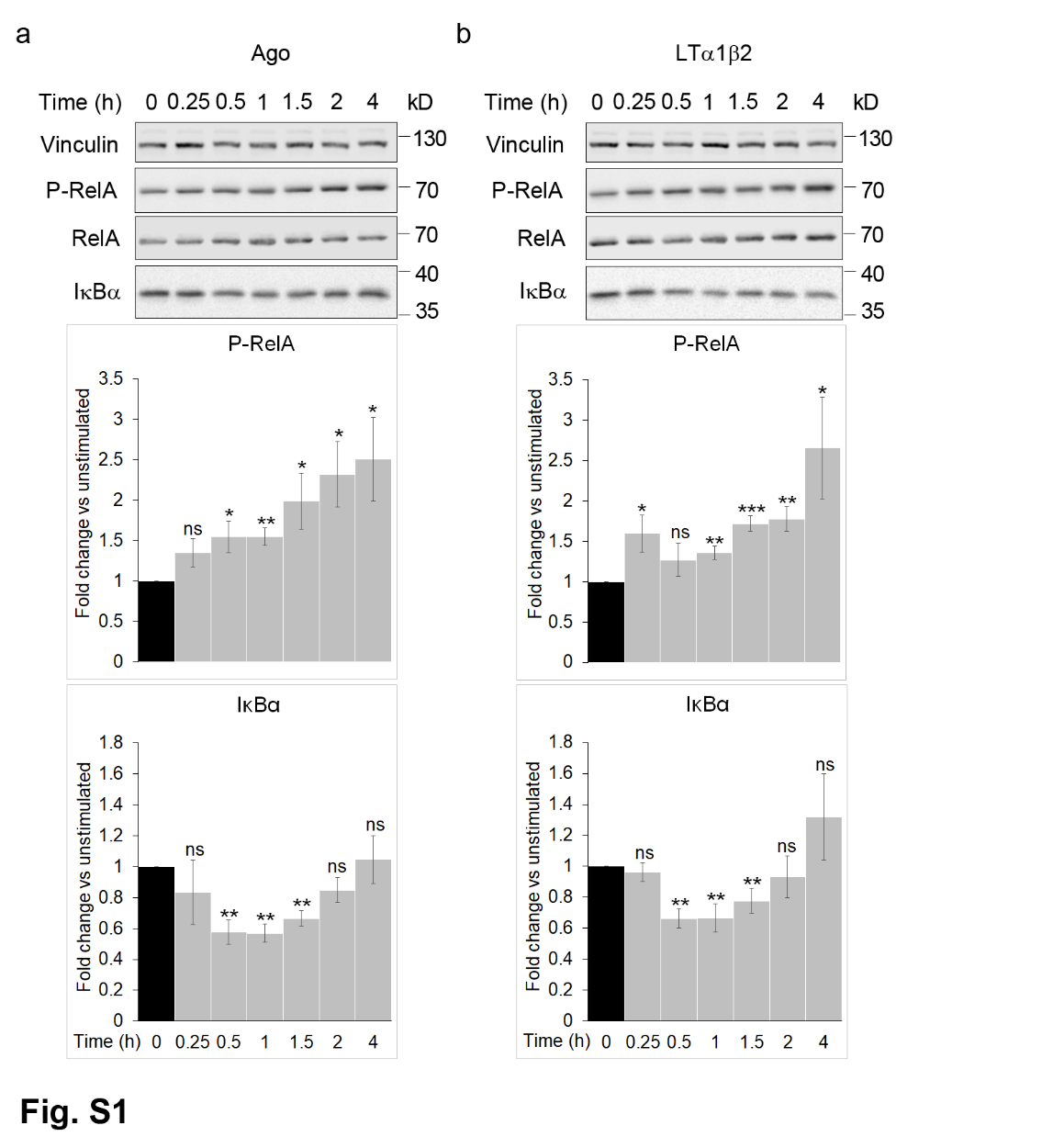
**

**Figure. S1. LTβR stimulation in A549 cells leads to activation of the canonical NF-κB pathway.**

**a, b** Lysates of A549 cells stimulated for the indicated times with LTβR agonist (Ago) **(a)** or lymphotoxin α1β2 (LTα1β2) **(b)** were analyzed by Western blotting with antibodies against the indicated proteins. Vinculin was used as a loading control. Graphs show densitometric analysis for P-RelA and IκBα from Western blotting (protein levels normalized to vinculin). Values are presented as fold change versus control - unstimulated cells (black bars). Data represent the means ± SEM, n≥5; ns - P>0.05; **P*≤0.05; ***P*≤0.01; ****P*≤0.001 by one sample t-test.

**
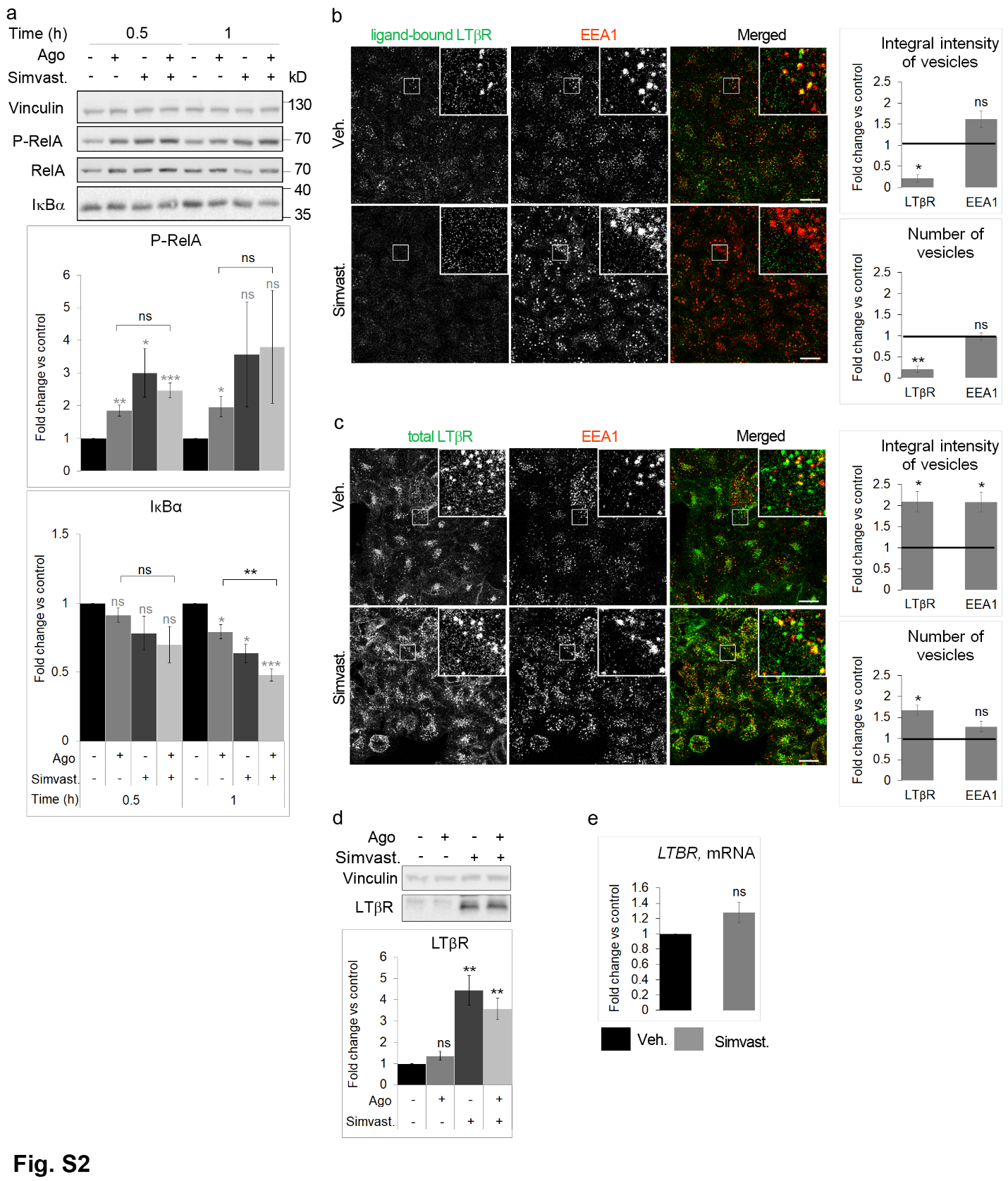
**

**Figure. S2.** **Inhibition of cholesterol synthesis by simvastatin activates NF-κB signaling and affects internalization of ligand-bound LTβR and its total cellular levels. a** Lysates of A549 cells preincubated for 48 h in delipidated medium containing simvastatin or vehicle and stimulated for 0.5 or 1 h with LTβR agonist (Ago) were analyzed by Western blotting with antibodies against the indicated proteins. Vinculin was used as a loading control. Graphs show densitometric analysis of the indicated proteins from Western blotting (protein levels normalized to vinculin). Values are presented as fold change versus controls - unstimulated and untreated cells (black bars). Data represent the means ± SEM, n=3; ns - *P*>0.05; ***P*≤0.01 by one sample t-test (in grey) or Student’s t-test (in black). **b** Immunofluorescence staining of ligand-bound LTβR and EEA1 in A549 cells upon 0.5 h stimulation with LTβR agonist in medium containing vehicle (Veh.) or simvastatin. Insets: magnified views of boxed regions in the main images. Scale bars, 20 µm. Graphs: analysis of integral intensity and number of LTβR- and EEA1-positive vesicles in cells treated as in B. Values are presented as fold change versus controls - vehicle-treated cells marked as a black line, set as 1. Data represent the means ± SEM, n=3. ns - *P*>0.05; **P*≤0.05; ***P*≤0.01 by one sample t-test. **c** Immunofluorescence staining of total LTβR and EEA1 in A549 cells treated for 48 h with either vehicle (Veh.) or simvastatin. Insets: Magnified views of boxed regions in the main images. Scale bars, 20 µm. Graphs: analysis of integral intensity and number of LTβR- and EEA1-positive vesicles in cells treated as in C. Values are presented as fold change versus controls - vehicle-treated cells marked as a black line, set as 1. Data represent the means ± SEM, n=3. ns - *P*>0.05; **P*≤0.05 by one sample t-test. **d** Lysates of A549 cells stimulated for 0.5 h with LTβR agonist (Ago) preincubated for 48 h with simvastatin or vehicle were analyzed by Western blotting with antibodies against the indicated proteins. Vinculin was used as a loading control. Graph show densitometric analysis of LTβR from Western blotting (protein levels normalized to vinculin). Values are presented as fold change versus controls – unstimulated and untreated cells (black bar). Data represent the means ± SEM, n=3; ns - *P*>0.05; ***P*≤0.01 by one sample t-test. **(e)** LTβR mRNA level in cells incubated with simvastatin. Values are presented as fold change versus controls – vehicle-treated cells (black bar). Data represent the means ± SEM, n=3. ns - P>0.05 by one sample t-test.

**
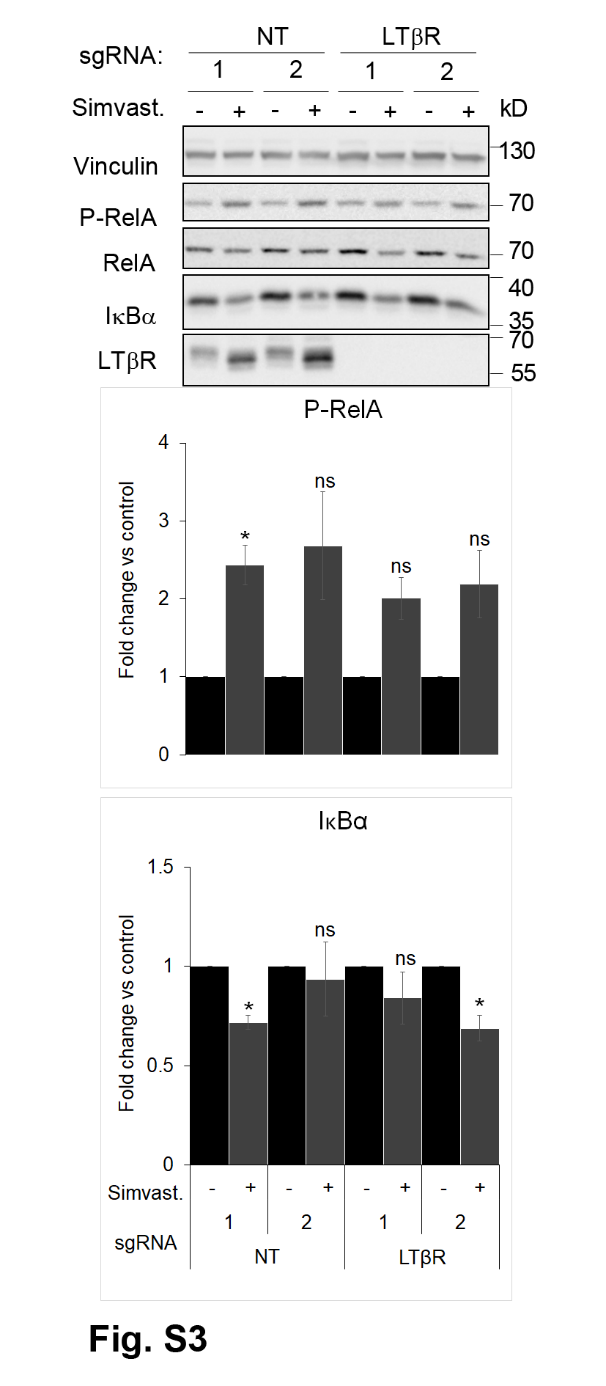
**

**Figure. S3.** **Inhibition of cholesterol synthesis by simvastatin activates NF-κB signaling in LTβR-independent manner.** A549 cells edited with the CRISPR/Cas9 method with two LTβR-targeting sgRNAs and two non-targeting (NT) sgRNAs were incubated with simvastatin or vehicle for 48 h. Cell lysates were analyzed by Western blotting with antibodies against the indicated proteins. Vinculin was used as a loading control. Graphs show densitometric analysis of the indicated proteins from Western blotting (protein levels normalized to vinculin). Values are presented as fold change versus controls - untreated cells (black bars). Data represent the means ± SEM, n=3; ns - *P*>0.05; **P*≤0.05 by one sample t-test.

**
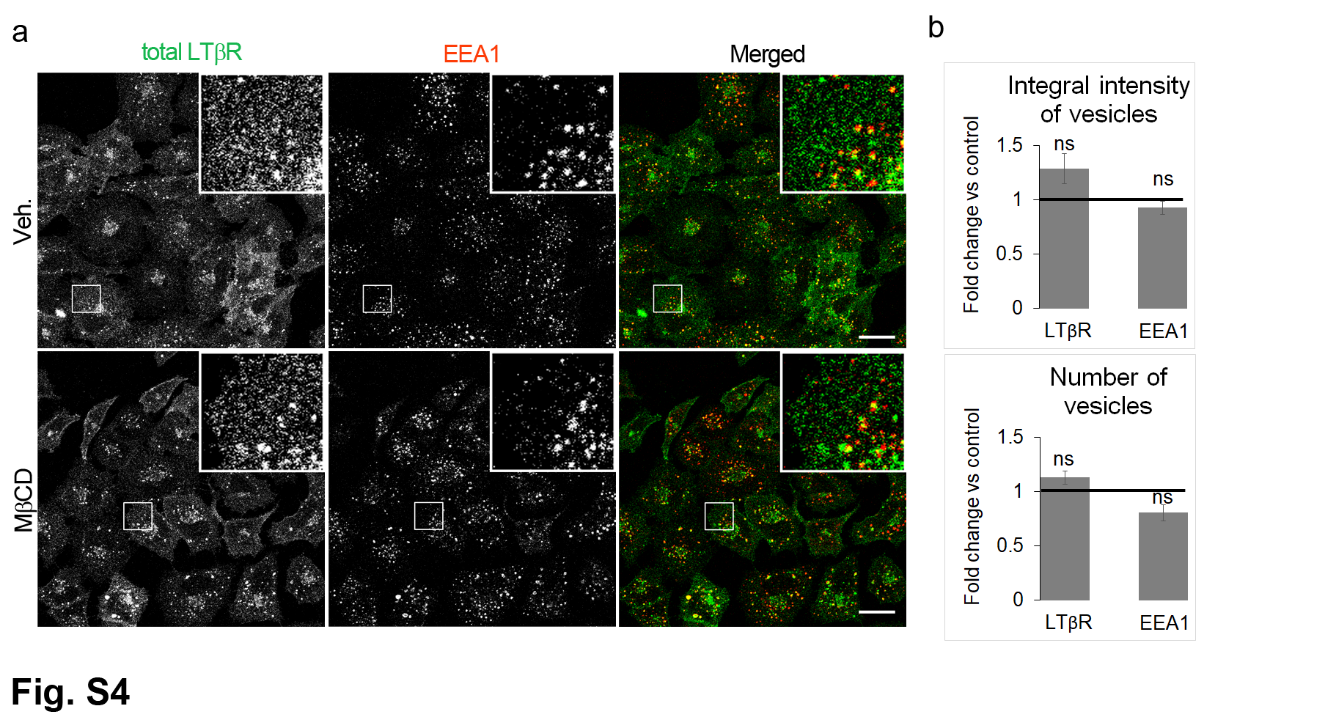
**

**Figure. S4.** **Cholesterol depletion by MβCD does not influence LTβR distribution within a cell. a** Immunofluorescence staining of total LTβR and EEA1 in A549 cells upon 1 h incubation with MβCD or vehicle (Veh.). Insets: magnified views of boxed regions in the main images. Scale bars, 20 µm. **b** Analysis of integral intensity and the number of LTβR- and EEA1-positive vesicles in cells treated as in A. Values are presented as fold change versus control - vehicle-treated cells marked as a black line, set as 1. Data represent the means ± SEM, n=3. ns - *P*>0.05 by one sample t-test.

**
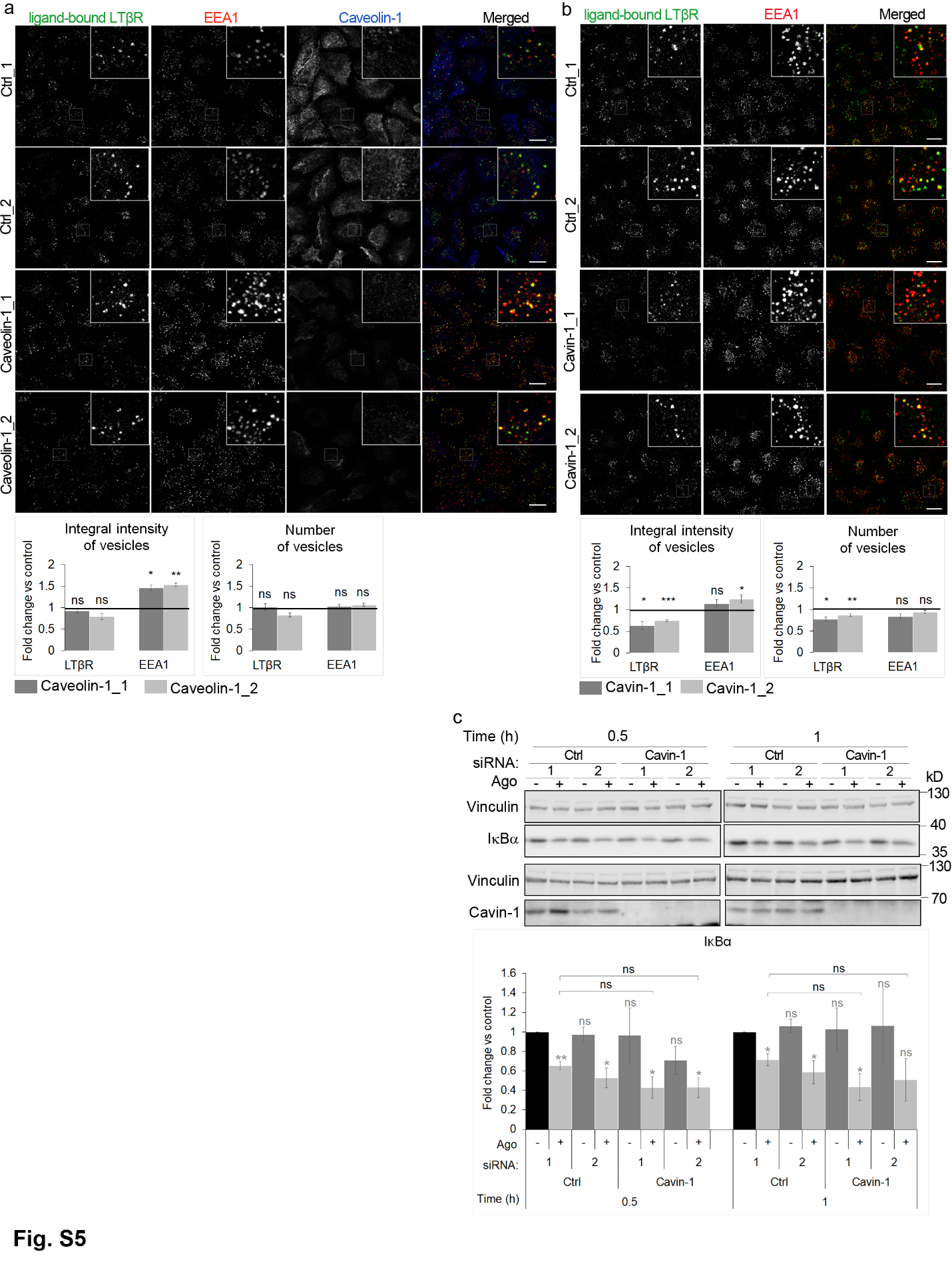
**

**Figure. S5. Perturbation in caveolae-dependent endocytosis does not affect LTβR-dependent signaling. a** Immunofluorescence staining of ligand-bound LTβR, EEA1 and caveolin-1 in A549 cells upon knockdown of caveolin-1 or in control (Ctrl) siRNA-transfected cells upon 0.5 h stimulation with Ago. Two siRNAs targeting caveolin-1 and two non-targeting siRNAs were used. Insets: magnified views of boxed regions in the main images. Scale bars, 20 µm. Graphs present analysis of integral intensity and the number of LTβR- and EEA1-positive vesicles in cells treated as in A. Values are presented as fold change versus cells transfected with non-targeting siRNA (Ctrl_1) marked as a black line, set as 1. Data represent the means ± SEM, n=3. ns - *P*>0.05; **P*≤0.05; ***P*≤0.01 by one sample t-test. **b** Immunofluorescence staining of ligand-bound LTβR and EEA1 in A549 cells upon knockdown of cavin-1 or in control (Ctrl) siRNA-transfected cells upon 0.5 h stimulation with Ago. Two siRNAs targeting cavin-1 and two non-targeting siRNAs were used. Insets: magnified views of boxed regions in the main images. Scale bars, 20 µm. Graphs present analysis of integral intensity and number of LTβR- and EEA1-positive vesicles in cells treated as in A. Values are presented as fold change versus cells transfected with non-targeting siRNA (Ctrl_1) marked as a black line, set as 1. Data represent the means ± SEM, n=5. ns - *P*>0.05; **P*≤0.05; ***P*≤0.01; ****P*≤0.001 by one sample t-test. **c** Lysates of A549 cells transfected with control (Ctrl) or cavin-1 targeting siRNAs (two oligonucleotides per gene) and stimulated with Ago for 0.5 and 1 h were analyzed by Western blotting with antibodies against the indicated proteins. Vinculin was used as a loading control. Graph shows densitometric analysis of IκBα from Western blotting (protein levels normalized to vinculin). Values are presented as fold change versus controls - unstimulated and untreated cells (black bars). Data represent the means ± SEM, n=4; ns - *P*>0.05; **P*≤0.05 by one sample t-test.

**
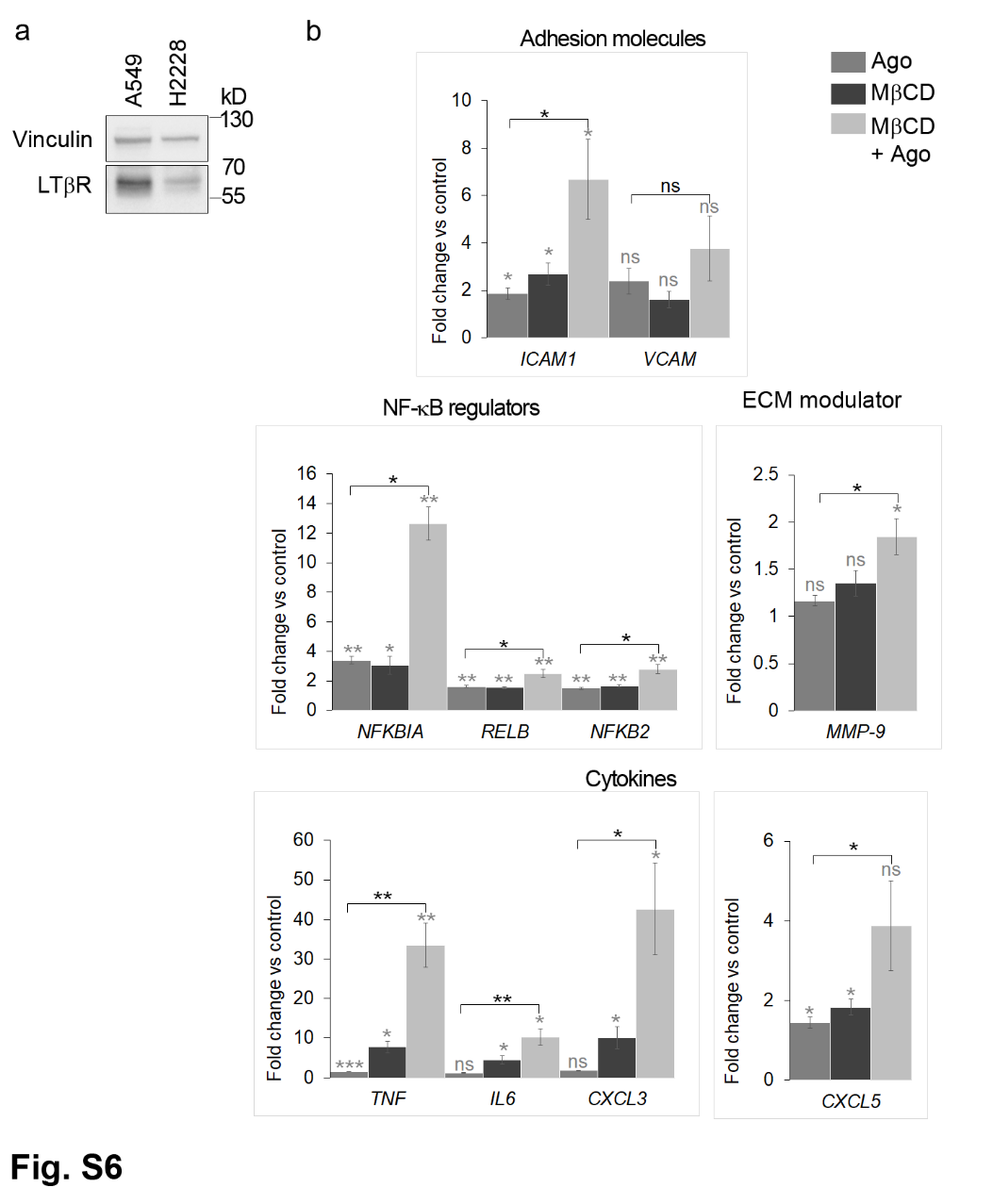
**

**Figure. S6. Cholesterol depletion enhances LTβR-triggered expression of NF-κB target genes in H2228 cell line. a** Lysates of H2228 cells were analyzed by Western blotting with antibodies against the indicated proteins. Vinculin was used as a loading control. **b** mRNA levels of the indicated NF-κB target genes in H2228 cells pretreated for 1 h with vehicle or MβCD and then stimulated for 2 h with LTβR agonist (Ago). Values are presented as fold change versus control – unstimulated and untreated cells, set as 1. Data represent the means ± SEM, n=4. ns - *P*>0.05; **P*≤0.05; ***P*≤0.01; ****P*≤0.001 by one sample t-test (in grey) or by Mann-Whitney or Student’s t-test (in black).
