# Supplementary material for: Cholesterol restricts lymphotoxin β receptor-triggered NF-κB signaling
Source: Cell Commun Signal. 2019 Dec 26;17:171. doi: 10.1186/s12964-019-0460-1 (PMC6933913; doi:10.1186/s12964-019-0460-1)
Supplement: Supplementary file 3 — Additional file 2: Table S1. Antibodies used for Western blotting. Table S2. Antibodies used for Immunofluorescence. Table 3. List of sequences of sgRNA used. Table 4. Sequences of primers used for qRT-PCR. Table 5. TaqMan® Gene Expression Assays used for qRT-PCR [file 12964_2019_460_MOESM3_ESM.docx]

**Additional file 2**

Table 1. Antibodies used for Western blotting

| **Antigen** | **Supplier** | **Cat#** | **source** | **Dilution** |
| --- | --- | --- | --- | --- |
| IκBα | Cell Signaling | 4814S | mouse | 1:1000 |
| RelA | Cell Signaling | 6956S | mouse | 1:1000 |
| P-RelA (S536) | Cell Signaling | 3033S | rabbit | 1:1000 |
| Vinculin | Sigma Aldrich | V9131 | mouse | 1:5000 |
| GAPDH | Santa Cruz | sc-25778 | rabbit | 1:2000 |
| LTβR | Santa Cruz | sc-8375 | goat | 1:500 |
| LTβR | Thermo Fisher Scientific | PA5-47028 | goat | 1:500 |
| TRAF2 | BD Laboratories | 558890 | mouse | 1:500 |
| NEMO | Santa Cruz | sc-8330 | rabbit | 1:1000 |
| p100/p52 | Cell Signaling | 4882S | rabbit | 1:1000 |
| Cavin-1 | Sigma Aldrich | AV36965 | rabbit | 1:1000 |
| Caveolin-1 | Thermo Fisher Scientific | PA1-064 | rabbit | 1:1000 |
| Ubiquitin | Santa Cruz | 3936S | mouse | 1:500 |

Table 2. Antibodies used for Immunofluorescence

| **Antigen** | **Supplier** | **Cat#** | **source** | **Dilution** |
| --- | --- | --- | --- | --- |
| EEA1 | BD Laboratories | 610457 | mouse | 1:1000 |
| EEA1 | Enzo Life Sciences | ALX-210-239 | rabbit | 1:400 |
| LTβR | R&D Systems | AF629 | goat | 1:100 |
| Caveolin-1 | Thermo Fisher Scientific | PA1-064 | rabbit | 1:500 |

Table 3. List of sequences of sgRNA used

| sgRNA name | Sequence |
| --- | --- |
| *LTBR-1* | CACCGGAAGGTGCCTCCATATGCGT (sense) |
| *LTBR-2* | CACCGCTCTGCAGGTGTGAGAACCA (sense) |
| *NT1* | CACCGCTGAAAAAGGAAGGAGTTGA |
| *NT2* | CACCGAAGATGAAAGGAAAGGCGTT |

Table 4. Sequences of primers used for qRT-PCR

| **Gene name** | **Forward primer** | **Reverse primer** |
| --- | --- | --- |
| *ACTB* | CAGGTCATCACCATTGGCAAT | TCTTTGCGGATGTCCACGT |
| *B2M* | GGAGGCTATCCAGCGTACTC | GAAACCCAGACACATAGCAATTC |
| *CCL20* | CTGGCTGCTTTGATGTCAGT | CGTGTGAAGCCCACAATAAA |
| *CXCL8* | GCTCTCTTGGCAGCCTTCCTGA | TTTCCTTGGGGTCCAGACAGAGC |
| *CXCL3* | ATCCCCCATGGTTCAGAAA | ACCCTGCAGGAAGTGTCAAT |
| *CXCL5* | GGAAAGATTTTGTTGTTGTT | AGTCACCTACAATTCAAGAC |
| *GM-CSF* | CATGATGGCCAGCCACTACAA | ACTGGCTCCCAGCAGTCAAAG |
| *ICAM1* | GGAGCCCGCTGAGGTCACGA | AGTCGCTGGCAGGACAAAGGT |
| *IL6* | AAGCCAGAGCTGTGCAGATGAGTA | TGTCCTGCAGCCACTGGTTC |
| *MMP9* | CTCTGGAGGTTCGACGTGAAGG | GGTCCTGGCAGAAATAGGCTT |
| *NFKBIA* | CGCCCAAGCACCCGGATACA | AGGGCAGCTCGTCCTCTGTGA |
| *NFKB2* | GCTGGAGGAGGCGGGCGTCTAA | GGGCTGGCTCCTTGGGTTCCA |
| *RELB* | GGAAAGACTGCACCGACGGCA | TTCAGGGACCCAGCGTTGTAGGG |
| *TNF* | GTGATCGGCCCCCAGAGGGA | TGAGGGTTTGCTACAACATGGGC |
| *VCAM* | CCGGATTGCTGCTCAGATTGGA | AGCGTGGAATTGGTCCCCTCA |

Table 5. TaqMan® Gene Expression Assays used for qRT-PCR

| **Gene name** | **TaqMan® Gene Expression Assays** |
| --- | --- |
| *ACTB* | Hs99999903_m1 |
| *GAPDH* | Hs02758991_g1 |
| *LTBR* | Hs00158922_M1 |
